# Effect of Autoinducer-2 Quorum Sensing Inhibitor on Interspecies Quorum Sensing

**DOI:** 10.3389/fmicb.2022.791802

**Published:** 2022-03-28

**Authors:** Kai Jiang, Yijie Xu, Bo Yuan, Yuandong Yue, Meihua Zhao, Rui Luo, Hao Wu, Lei Wang, Yuanyuan Zhang, Junhai Xiao, Feng Lin

**Affiliations:** ^1^School of Life Sciences, Jilin University, Changchun, China; ^2^Key Laboratory for Molecular Enzymology and Engineering, The Ministry of Education, School of Life Sciences, Jilin University, Changchun, China; ^3^National Engineering Research Center for Strategic Drugs, Beijing Institute of Pharmacology and Toxicology, Beijing, China; ^4^State Key Laboratory of Toxicology and Medical Countermeasures, Beijing Institute of Pharmacology and Toxicology, Beijing, China; ^5^No. 971 Hospital of People’s Liberation Army Navy, Qingdao, China

**Keywords:** *Pseudomonas aeruginosa* PAO1, *Staphylococcus aureus* ATCC 25923, AI-2 quorum sensing, ethylene diamine triacetic acid group, interspecies

## Abstract

Bacterial drug resistance caused by overuse and misuse of antibiotics is common, especially in clinical multispecies infections. It is of great significance to discover novel agents to treat clinical bacterial infections. Studies have demonstrated that autoinducer-2 (AI-2), a signal molecule in quorum sensing (QS), plays an important role in communication among multiple bacterial species and bacterial drug-resistance. Previously, 14 AI-2 inhibited compounds were selected through virtual screening by using the AI-2 receptor protein LuxP as a target. Here, we used *Vibrio harveyi* BB170 as a reporter strain for the preliminary screening of 14 inhibitors and compound Str7410 had higher AI-2 QS inhibition activity (IC_50_ = 0.3724 ± 0.1091 μM). Then, co-culture of *Pseudomonas aeruginosa* PAO1 with *Staphylococcus aureus* ATCC 25923 was used to evaluate the inhibitory effects of Str7410 on multispecies infection *in vitro* and *in vivo*. *In vitro*, Str7410 significantly inhibited the formation of mixed bacterial biofilms. Meanwhile, the combination of Str7410 with meropenem trihydrate (MEPM) significantly improved the susceptibility of mixed-species-biofilm cells to the antibiotic. *In vivo*, Str7410 significantly increased the survival rate of wild-type *Caenorhabditis elegans* N2 co-infected by *P. aeruginosa* PAO1 and *S. aureus* ATCC 25923. Real-time quantitative PCR analysis showed that Str7410 reduced virulence factor (pyocyanin and elastase) production and swarming motility of *P. aeruginosa* PAO1 by downregulating the expression of QS-related genes in strain PAO1 in co-culture with *S. aureus* ATCC 25923. Compound Str7410 is a candidate agent for treating drug-resistant multispecies infections. The work described here provides a strategy for discovering novel antibacterial drugs.

## Introduction

Most bacterial diseases are caused by infection with multiple species of bacteria, for example, cystic fibrosis (CF) and chronic wounds (Harrison, 2007; DeLeon et al., 2014; Blanchard and Waters, 2019). On co-infection by multiple bacteria, different strains interact with each other and contribute to the pathogenesis of the disease. The pathogenic mechanisms resulting from the interaction of multiple microbes are often different from those of individual species (Baldan et al., 2014).

*Pseudomonas aeruginosa* and *Staphylococcus aureus* are the two most important pathogens in *CF* and chronic wounds (DeLeon et al., 2014; Blanchard and Waters, 2019). Interactions between *P. aeruginosa* and *S. aureus* have been the focus of several studies on multiple-strain infections. Notably, *P. aeruginosa* significantly increased the production of *S. aureus* biofilms and resistance of *S. aureus* to vancomycin on co-culture *in vitro* (Yang et al., 2011; Orazi and O’Toole, 2017). In a wound model co-infected by *P. aeruginosa* and *S. aureus*, the resistance of the bacteria to antibiotics was significantly increased, and the toxicity of *P. aeruginosa* was also increased (Dalton et al., 2011; DeLeon et al., 2014). Moreover, after co-infection by *P. aeruginosa* and *S. aureus*, the healing of pig epithelial cell wounds (Pastar et al., 2013) and mouse wounds (Korgaonkar et al., 2013) was significantly delayed, and *S. aureus* promoted the pathogenicity of *P. aeruginosa*. Korgaonkar et al., 2013 showed that peptidoglycan produced by *S. aureus* can significantly increase the lethality of *P. aeruginosa* toward *Drosophila* and promote the production of *P. aeruginosa* virulence factors such as pyocyanin and elastase. In the sputum of *CF* patients, *P. aeruginosa* and other bacteria, such as *Streptococcus* and *Staphylococcus*, can communicate with each other through the autoinducer-2 (AI-2) quorum sensing (QS) system (Duan et al., 2003). This indicates that different bacteria in multispecies infections can increase in pathogenicity through QS.

Quorum sensing is a signaling mechanism that regulates the life activities of bacteria by transmitting signals through the synthesis, release, and acceptance of autoinducers (AIs). Bacteria sense the density of the surrounding population *via* AI molecules, and this regulates gene expression (Gokalsin et al., 2017). AI-2 is an intra- and interspecies signal molecule “a common language” for bacterial interaction. AI-2 QS was first discovered and characterized in the Gram-negative marine bacterium *Vibrio harveyi* (Bassler et al., 1994). Bioluminescence of *V. harveyi* is regulated by QS. *V. harveyi* produces AI-2 signal molecule through LuxS protease and after AI-2 binds to LuxP receptor, the LuxP–AI-2 complex then interacts with LuxQ phosphokinase in the membrane, and finally LuxR receptor protein regulates the production of bioluminescence (Roy et al., 2011). Therefore, *V. harveyi* is often used as a test species for laboratory AI-2 QS inhibitors (QSIs) research (Lowery et al., 2008; Collins et al., 2015). Research found >40 Gram-positive and Gram-negative bacterial species can communicate using AI-2 as a signaling molecule (Mok et al., 2003). AI-2-mediated QS plays a critical role in the interaction among multiple strains, and it has also been shown that AI-2 is closely related to the formation of mixed biofilms and gene regulation among multiple strains (Roy et al., 2011).

As a Gram-positive pathogen, *S. aureus* has two types of QS system. One is the autoinducing peptide signaling molecule-mediated *agr* system, and the other is the LuxS/AI-2 system (Sifri, 2008). *Staphylococcus aureus* is able to produce AI-2 signaling molecule, which is regulated by the *luxS* gene. However, no potential AI-2 receptor (such as the LuxPQ receptor of *Vibrio harveyi* or the LsrABC transporter of *Salmonella enterica* serovar Typhimurium) has been found by searching for established AI-2 receptors in *S. aureus* genomes (Zhao et al., 2010). The LuxS/AI-2 system can negatively regulate the formation of *S. aureus* biofilms (Ma et al., 2017), and loss of the *luxS* gene leads to a decrease in susceptibility to cell wall synthesis inhibitor antibiotics (Wang et al., 2019). Because of the dual function of LuxS and the absence of genomic evidence of established AI-2 receptors, the AI-2 QS function in *S. aureus* needs further study (Zhao et al., 2010).

*Pseudomonas aeruginosa* lacks the *luxS* gene and cannot produce AI-2 signaling molecule. However, *P. aeruginosa* regulates the production of a variety of virulence factors and biofilms through the *las*, *rhl*, *pqs*, and *iqs* QS systems, which destroys tissues and induces inflammation, leading to impaired immune mechanisms, in an infected patient (Van Delden and Iglewski, 1998). Therefore, studies in *P. aeruginosa* have focused on QSIs that target QS mediated by acyl-homoserine lactone (AHL) signaling molecules (Chbib, 2020; Jiang et al., 2020). Nevertheless, *P. aeruginosa* can sense the AI-2 signaling molecule produced by other bacteria, such as *Escherichia coli* (Roy et al., 2011), *Salmonella typhimurium* (Roy et al., 2011), *Streptococcus mitis* (Wang et al., 2016b), and *S. aureus* (Hotterbeekx et al., 2017), which regulates the production of virulence factors and biofilms and increases its pathogenicity (Li et al., 2015; Wang et al., 2016b). Recent studies have shown that C_1_-alkyl AI-2 analogs reduced *V. harveyi* QS-associated bioluminescence (Lowery et al., 2009), and analogs of 4,5-dihydroxy-2,3-pentanedione (DPD), a precursor substance of AI-2 signaling molecule, like butyl and pentyl-DPD, were shown to inhibit pyocyanin production by *P. aeruginosa* by 50% (Ganin et al., 2009). Inhibiting the AI-2 QS system using a QSI is a new strategy to treat bacterial infections caused by *P. aeruginosa* and *S. aureus*.

In our previous study, 14 AI-2 QSI compounds were selected through virtual screening. In this study, *V. harveyi* BB170 was used as a reporter strain for the preliminary screening. Compound Str7410, which had the highest AI-2 QS inhibition activity, was chosen for further research. We then co-cultured *P. aeruginosa* PAO1 with *S. aureus* ATCC 25923 to analyze the inhibitory effects of compound Str7410 on multispecies infections *in vitro* and *in vivo*. The effects of the compound on biofilm formation and of the combination of Str7410 with antibiotics on drug resistance were determined *in vitro*. We tested the effects of Str7410 on *P. aeruginosa* PAO1 virulence factor (pyocyanin and elastase) production, swarming motility, and the expression of QS-related genes in co-culture of *P. aeruginosa* and *S. aureus*. We used *Caenorhabditis elegans* N2 as an *in vivo* model to test the effects of Str7410 on survival rates on co-infection of the nematode with *P. aeruginosa* and *S. aureus*. Finally, we preliminarily evaluated the inhibitory mechanism of interspecies QS by the compound. This study provides a new strategy for the treatment of clinical multispecies infections.

## Materials and Methods

### Synthesis of Compound Str7410

**Scheme 1:**
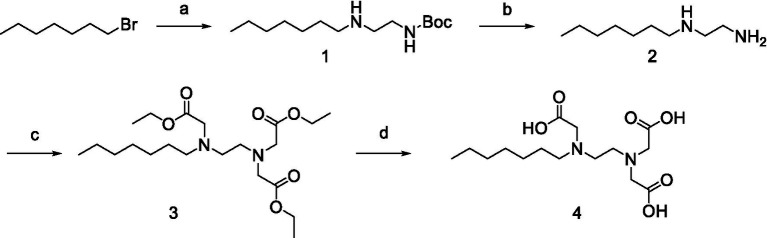
Synthetic route toward the target compound Str7410. ^ɑ^Reagents and conditions. **(A)** *N*-tert-butoxycarbonyl-1,2-ethylenediamine, K_2_CO_3_, MeCN, 65°C, and 63% yield; **(B)** trifluoroacetic acid, CH_2_Cl_2_, room temperature, and 100% yield; and **(C)** K_2_CO_3_, ethyl 2-bromoacetate, MeCN, 45°C, and 56% yield; **(D)** concentrated hydrochloric acid, 100°C, and 54% yield.

All the chemical reagents used in the synthesis reactions were analytical-grade and available from commercial resources without further purification. All the reactions were monitored by analytical thin-layer chromatography (TLC) using silica gel TLC plates (GF254). Silica gel (200–300 mesh) was used for chromatography. The ^1^H and ^13^C nuclear magnetic resonance (NMR) spectroscopy were recorded on a JEOLECA400 spectrometer, with TMS as an internal standard at ambient temperature. All coupling constants were reported in Hertz. Proton coupling patterns were described as singlet (s), doublet (d), triplet (t), quartet (q), multiplet (m), and broad (br). High-resolution mass spectra (HRMS) were obtained by electrospray ionization (ESI) using an Agilent TOF G6230A mass spectrometer. The synthesis of compound Str7410 was shown in Scheme 1. Intermediates 1, 2, and 3 were prepared by the following routes.

### Synthesis of Tert-Butyl (2-(Heptylamino)Ethyl)Carbamate (1)

To a solution of *N*-tert-butoxycarbonyl-1,2-ethylenediamine (4.49 g, 28.0 mmol) in CH_3_CN (60 ml) was added K_2_CO_3_ (1.55 g, 11.2 mmol), then the mixture was heated to 65°C. At this temperature, 1-bromoheptane (1 g, 5.6 mmol) in CH_3_CN (10 ml) was added dropwise to the mixture. The mixture was stirred at 80°C for 6 h and cooled to room temperature. The solvent was concentrated *in vacuo*. The residue was dissolved in CHCl_3_, washed with brine, dried over Na_2_SO_4_, and concentrated *in vacuo*. The residue was purified by flash silica gel chromatography (1% triethylamine, petroleum ether: ethyl acetate = 1: 3) to afford 1 as colorless oil (0.91 g, 63%). ^1^H NMR [400 MHz, dimethylsulfoxide (DMSO)-d_6_] δ 6.69 (*t*, *J* = 6.8 Hz, 1H), 2.98 (*q*, *J* = 6.2 Hz, 2H), 2.53–2.48 (*m*, 2H), 2.46 (*t*, *J* = 7.0 Hz, 2H), 1.37 (*s*, 11H), 1.25 (*s*, 8H), and 0.86 (*t*, *J* = 6.8 Hz, 3H). ^13^C NMR (101 MHz, DMSO) δ 156.14, 77.94, 49.52, 31.83, 30.10, 29.21, 28.75, 27.29, 22.59, and 14.46. HRMS (ESI) of C_14_H_30_N_2_O_2_ [M + H]^+^ calcd., 259.2381; found 259.2380.

### Synthesis of *N*^1^-Heptylethane-1,2-Diamine (2)

To a cold (0°C) solution of trifluoroacetic acid (2.5 ml) and CH_2_Cl_2_ (7.5 ml) was added tert-butyl (2-(heptylamino)ethyl)carbamate (0.5 g, 1.9 mmol), then the mixture was warmed to room temperature and stirred for 2 h. The solution was concentrated *in vacuo* and used directly in the next step without further purification.

### Synthesis of Diethyl 2,2'-((2-((2-Ethoxy-2-Oxoethyl)(Heptyl)Amino)Ethyl)Azanediyl)Diacetate (3)

To a solution of *N*^1^-heptylethane-1,2-diamine (0.3 g, 1.9 mmol) in CH_3_CN (40 ml) was added K_2_CO_3_ (1.05 g, 7.6 mmol) and ethyl 2-bromoacetate (0.98 g, 5.9 mmol). Then, the mixture was stirred at 45°C for 3 h and cooled to room temperature. The solvent was concentrated *in vacuo*. The residue was dissolved in CHCl_3_, washed with brine, dried over Na_2_SO_4_, and concentrated *in vacuo*. The residue was purified by flash silica gel chromatography (1% triethylamine, CH_2_Cl_2_: CH_3_OH = 20: 1) to afford 3 as colorless oil (0.44 g, 56%). ^1^H NMR (400 MHz, DMSO-d_6_) δ 4.09 (*t*, *J* = 7.1 Hz, 6H), 3.57 (*s*, 4H), 3.37 (*s*, 2H), 2.93 (*d*, *J* = 5.0 Hz, 2H), 2.89 (*d*, *J* = 4.9 Hz, 2H), 2.86–2.81 (*m*, 2H), 1.50 (*m*, 2H), 1.27 (*s*, 8H), 1.21 (*t*, *J* = 7.1 Hz, 9H), and 0.87 (*d*, *J* = 7.1 Hz, 3H). ^13^C NMR (101 MHz, DMSO) δ 171.47, 169.40, 60.46, 56.23, 55.06, 54.58, 52.49, 50.32, 31.64, 28.90, 26.73, 25.54, 22.53, 14.60, and 14.43. HRMS (ESI) of C_21_H_40_N_2_O_6_ [M + H]^+^ calcd., 417.2959; found 417.2960.

### Synthesis of 2,2'-((2-((Carboxymethyl)(Heptyl)Amino)Ethyl)Azanediyl)Diacetic Acid (4)

Diethyl 2,2'-((2-((2-ethoxy-2-oxoethyl)(heptyl)amino)ethyl)azanediyl) diacetate (0.2 g, 0.5 mmol) was added to concentrated hydrochloric acid (15 ml). The mixture was heated to 100°C and stirred for 12 h. Then, the residue was cooled to room temperature and concentrated *in vacuo*. The residue was added to concentrated hydrochloric acid (15 ml), and the mixture was stirred at 100°C for 12 h and concentrated *in vacuo*. The residue was recrystallized with ethanol (5 ml) and methyl tert-butyl ether (MTBE, 45 ml), filtered, and washed with MTBE to afford 4 as white solid (86.2 mg, 54%). ^1^H NMR (400 MHz, DMSO-d_6_) δ 4.23 (*s*, 2H), 3.63 (*s*, 4H), 3.37 (*t*, *J* = 5.6 Hz, 2H), 3.26–3.19 (*m*, 2H), 3.16 (*t*, *J* = 5.9 Hz, 2H), 1.71–1.59 (*m*, 2H), 1.26 (*s*, 8H), and 0.89–0.83 (*m*, 3H). ^13^C NMR (101 MHz, DMSO) δ 172.64, 168.12, 55.11, 54.97, 53.39, 51.70, 49.28, 31.51, 28.67, 26.34, 23.68, 22.49, and 14.44. HRMS (ESI) of C_15_H_28_N_2_O_6_ [M + H]^+^ calcd., 333.2020; found 333.2019.

### Bacterial Strains and Growth Media

*Vibrio harveyi* BB170 (ATCC BAA-1117), *S. aureus* ATCC 25923, *P. aeruginosa* PAO1, and *E. coli* OP50 were used in this study. AB medium contained 0.3 M NaCl, 0.05 M MgSO_4_, and 2% acid-hydrolyzed casein (without vitamins), adjusted to pH 7.5 with 1 M KOH. After autoclaving at 121°C, 10 ml of 1 M potassium phosphate buffer (K_2_HPO_4_/KH_2_PO_4_, pH 7.0), 10 ml of 0.1 M sterile arginine solution (L-Arg), and 20 ml of 50% sterile glycerol were added to the medium (per 1 L). LB medium contained 1% tryptone, 1% NaCl, and 0.5% yeast extract. TSB medium contained 1.5% tryptone, 0.5% soy peptone, and 0.5% NaCl (pH = 7.2 ± 0.2). PB medium contained 2% peptone, 1% K_2_SO_4_, and 0.3% MgCl_2_. PTSB medium contained 5% peptone, 1.7% tryptone, 0.5% NaCl, 0.25% K_2_HPO_4_, 0.25% glucose, and 0.3% soy peptone. PGS-agar plates contained 1% peptone, 0.15 M sorbitol, 1% NaCl, 1% glucose, and 1.7% agar. Swimming solid medium contained 0.8% nutrient broth, 0.5% glucose, and 0.3% agar. Swarming solid medium contained 0.8% nutrient broth, 0.5% glucose, and 0.5% agar.

Test compounds were dissolved in DMSO to a stock concentration of 65 mM. Antibiotics [tobramycin base, meropenem trihydrate (MEPM), ceftazidime, amikacin, colistin sulfate, and ciprofloxacin] were purchased from Sigma and stored at 6.4 mg/ml at −20°C.

### Half-Maximal Inhibitory Concentration of Compounds in Assay of *Vibrio harveyi* BB170 QS

The concentration of compounds that resulted in 50% maximum *V. harveyi* BB170 bioluminescence (the IC_50_ value) was tested as described previously (Collins et al., 2015). *Vibrio harveyi* BB170 was grown in AB medium (14 h, 30°C) to OD_600 nm_ = 1.5. The cells were diluted into fresh AB medium (1:2,500) and added (100 μl/well) to serially diluted test compounds in AB medium (100 μl/well); the final DMSO concentration was 0.5%. The plate was incubated at 30°C for 8 h, and then the bioluminescence (OD_460 nm_) and cell density (OD_600 nm_) were measured. The values of OD_460 nm_ and OD_600 nm_ were normalized and IC_50_ values were calculated using GraphPad Prism 5 software.

### Growth Assays

*Pseudomonas aeruginosa* PAO1 and *S. aureus* ATCC 25923 were inoculated into LB medium, respectively, and cultured at 37°C at 150 rpm until OD_600 nm_ = 0.5. Then, the cultures were diluted with fresh LB medium to OD_600 nm_ = 0.05. Next, the bacteria were cultured in the presence of compound Str7410 (80, 40, 20, 10, and 1 μM), and OD_600 nm_ was measured at intervals of 2 h for up to 24 h using a spectrophotometer (UV-1800, Bio-Rad Smart Spec Plus, United States). All experiments were performed three times, independently (Wang et al., 2016b).

### Biofilm Quantification Assays

Quantitative analysis of biofilms used crystal violet assay (Armbruster et al., 2016). First, we used fresh LB medium to dilute *P. aeruginosa* PAO1 and *S. aureus* ATCC 25923 cultures in the logarithmic growth phase to OD_600 nm_ = 0.05, and then mixed them in equal proportions in the same volume and added them to a 96-well plate (100 μl/well, three parallel wells for each group). The final test compound concentrations were 80, 40, 20, 10, and 1 μM. The 96-well plate was incubated at 37°C for 24 h, then the bacterial culture was removed, and the plate was washed three times with phosphate-buffered saline (PBS) and dried. Next, 0.1% (w/v) crystal violet solution was added for 10 min. After washing three times with PBS, the crystal violet-stained biofilms were air-dried. To quantify the biofilm biomass, the crystal violet was removed by adding 150 μl of 33% glacial acetic acid, and OD_595 nm_ of the solubilized dye was determined using a Biotek multifunction microplate reader. The biofilm inhibition rate was defined as (OD_595 blank_ − OD_595 sample_)/OD_595 blank_ × 100%. Each crystal violet assay was run in triplicate, with a minimum of three replicates per assay.

### Determination of Susceptibility to Antibiotics

The determination of minimal inhibitory concentrations (MICs) was performed as described previously (Peeters et al., 2009; Guo et al., 2016). *Pseudomonas aeruginosa* PAO1 and *S. aureus* ATCC 25923 were, respectively, cultured in LB medium to OD_600 nm_ = 0.8. *Pseudomonas aeruginosa* PAO1 alone or in co-culture with *S. aureus* ATCC 25923 (mixed in equal proportions) were diluted 1,000-fold with fresh LB medium and added to a 96-well plate. According to the broth dilution method, Antibiotics concentrations ranged from 64 to 0.125 μg/ml. Finally, the plates were incubated at 37°C for 18 h, and OD_590 nm_ was determined using a Biotek multifunction microplate reader.

Determination of minimum biofilm inhibitory concentrations (MBICs) of antibiotics (Wang et al., 2016a). Based on the above method, 100 μl of diluted bacteria was added to each well of a 96-well plate and cultured at 37°C for 48 h. After removing the bacterial culture, the biofilms were washed twice using PBS. Antibiotics and fresh LB medium were added to the 96-well plate, which was incubated at 37°C for 24 h. The lowest drug concentration without turbidity well is MBIC value of antibiotics.

For the minimum biofilm eradication concentrations (MBECs) of compound for biofilm cells (Tsukatani et al., 2016, 2020), the 96-well plate containing 100 μl diluted bacterial solution of each well was incubated at 37°C for 24 h. After removing the bacterial culture, the biofilms were washed three times using PBS, then 180 μl reaction solution of 2-fold serial dilutions of compound (range 1,024–1 μg/ml) in fresh LB medium added to 96-well plate. After 20 h challenge at 37°C, the reaction solution were removed and washed three times with sterile PBS, then 150 μl fresh LB medium added to each well of 96-well plate and incubated for a further 24 h at 37°C. After removing the LB medium, the biofilms were washed three times using PBS and dried, and then 150 μl 0.1% (w/v) crystal violet solution was added for 10 min. After washing three times with PBS, adding 150 μl of 33% glacial acetic acid to 96-well plate, and the OD_460 nm_ was measured using a Biotek multifunction microplate reader. The MBEC represents the lowest concentration of constituents for which the biofilm eradication activity was >99%. Biofilm eradication activity (%) was determined using the following formula:


$$\begin{array}{l} \mathrm{Biofilm eradication}\\ \;\;\;\;\;\;\;\;\;\;\;\;\;\;\;\;\;\;\mathrm{activity}\;\left(\%\right)=\left({\mathrm{OD}}_{460\;\mathrm{control}}-{\mathrm{OD}}_{460\;\mathrm{blank}}\right)-\\ \;\;\;\;\;\;\;\;\;\;\;\;\;\;\;\;\;\;\;\;\;\;\;\;\;\;\;\;\;\;\;\;\;\;\;\;\;\;\;\;\;\;\;\;\;\;\;\;\;\;\;\;\;\;\;\left({\mathrm{OD}}_{460\;\mathrm{sample}}-{\mathrm{OD}}_{460\;\mathrm{blank}}\right)/\\ \;\;\;\;\;\;\;\;\;\;\;\;\;\;\;\;\;\;\;\;\;\;\;\;\;\;\;\;\;\;\;\;\;\;\;\;\;\;\;\;\;\;\;\;\;\;\;\;\;\;\;\;\;\;\;\left({\mathrm{OD}}_{460\;\mathrm{control}}-{\mathrm{OD}}_{460\;\mathrm{blank}}\right)\times 100\% \end{array}$$

Treatment of biofilms and quantification of cells was performed as follows. Dual-species biofilms were grown in 96-well plate. The liquid medium was decanted every 2 days leaving the attached cells at the bottom of the plate, and fresh LB medium was added (Armijo et al., 2020). After 6 days, the liquid culture was removed and the biofilms were slowly washed twice with PBS. In the test compound + antibiotic group, the test compound (1 μl, 10 μM final concentration), antibiotic (1 μl of MEPM, 0.5 μg/ml final concentration), and LB medium (98 μl) were added to each well. In the test compound or antibiotic alone groups, 99 μl of LB medium and 1 μl of test compound or MEPM were added, respectively. In the antibiotic + sonication group, bacteria in the biofilm were detached by sonication (40 kHz, 300 W, and 5 min) before adding 99 μl of LB medium and 1 μl of MEPM. In the control group, 100 μl of LB medium was added. After incubation for 3.5 h, biofilm cells were detached by sonication (40 kHz, 300 W, and 5 min) and the number of colony-forming units was determined by plating the resulting suspension (Brackman et al., 2011; Guo et al., 2016).

### *Caenorhabditis elegans* Survival Assay

*Pseudomonas aeruginosa* PAO1 and *S. aureus* ATCC 25923 were cultured to OD_600 nm_ = 2.0 at 37°C in TSB medium, respectively. Then, after mixing in equal proportions, 20 μl of culture were spread on PGS-agar plates containing antibiotic or test compound. The plates were placed in an incubator at 37°C and cultured for 18 h to allow the formation of a bacterial lawn. Then, 50 synchronized nematodes (L4 stage of wild-type *C. elegans* N2) were selected and placed on a plate. The plates were incubated at 20°C and the number of living and dead nematodes was counted every 24 h using a stereomicroscope. A nematode was defined as dead when it no longer responded to touch. Any nematodes that died as a result of getting stuck to the wall of the plate were excluded from the analysis. Survival curves were drawn for analysis (Rajkumari et al., 2018).

### Virulence Factor Quantification

Pyocyanin was quantified as follows: *P. aeruginosa* PAO1 and *S. aureus* ATCC 25923 were, respectively, cultured in LB medium at 37°C, 150 rpm, to the logarithmic growth phase. After mixing in equal proportions, the culture was diluted tenfold with fresh PB medium. Bacterial cultures (5 ml) with and without the test compound (40, 20, and 10 μM) were incubated at 37°C and 150 rpm for 16 h. To extract pyocyanin, the culture was centrifuged at 1,665 × *g* for 10 min, and the supernatant was collected and extracted with 3 ml of chloroform. Then, 1 ml of 0.2 M HCl was mixed with the chloroform layer, and the upper (aqueous) phase was collected by centrifugation at 1,665 × *g* for 10 min. The absorbance was measured at 520 nm (Essar et al., 1990). The inhibition rate of pyocyanin production was calculated as (OD_520 blank_ − OD_520 sample_)/OD_520 blank_ × 100%.

Elastase was determined using the Elastin-Congo red assay with modifications, and the final absorbance was read at 495 nm (Pearson et al., 1997). *Pseudomonas aeruginosa* PAO1 and *S. aureus* ATCC 25923 were, respectively, cultured in PTSB medium at 37°C and 150 rpm to the logarithmic growth phase. After mixing in equal proportions, co-cultures were divided into the experimental group and the control group, and then incubated at 37°C and 150 rpm for 18 h. Then, the cultures were centrifuged at 9,590 × *g* for 5 min and the supernatant was collected. The bacteria were filtered through a 0.22-μm pore-size filter. Then, 200 μl of the filtrate was added to 1 ml of Elastin-Congo red reaction solution [20 mg/ml Congo red, 0.1 M Tris–HCl (pH 7.2), and 1 mM CaCl_2_], and the mixture was shaken for 18 h at 37°C. Stop-reaction solution (ethylenediaminetetraacetic acid, 0.1 ml, and 0.12 M) was added and the mixture was incubated on ice for 5 min, and then centrifuged at 9,590 × *g* for 10 min. The supernatant was collected and OD_495nm_ was determined.

### Swarming Motility Assay

*Pseudomonas aeruginosa* PAO1 was shaken in LB medium at 37°C until OD_600 nm_ = 1.0. Bacterial culture (1 μl) was inoculated into the center of cooled swimming solid medium and further cultured at 30°C for 16 h. *Staphylococcus aureus* ATCC 25923 was shaken at 37°C in LB medium to OD_600 nm_ = 0.5. The culture was centrifuged at 9,590 × *g* for 5 min, and the supernatant was filtered (0.22-μm pore-size). The filtered supernatant was added to thawed swarming solid medium at a ratio of 1:10, mixed, poured into a plate, and left to stand for 8 h to dry. Finally, colonies were picked from the *P. aeruginosa* PAO1 swimming solid medium, added to the center of the swarming solid plate, and cultured at 30°C for 16 h (Tremblay and Deziel, 2008; Pallett et al., 2019).

### Quantitative Reverse Transcriptase PCR

*Pseudomonas aeruginosa* PAO1 and *S. aureus* ATCC 25923 cultured overnight for 16 h were diluted with fresh LB medium to OD_600 nm_ = 0.05. After mixing in equal proportions, they were divided into a control group and an experimental group (with test compound added) and grown at 37°C and 150 rpm for 10 h. *Pseudomonas aeruginosa* PAO1 was also grown in monoculture. After centrifugation (9,590 × *g*, 5 min) to retain the bacterial cells, a one-step cDNA kit was used [5× All-In-One RT MasterMix (With Excellent Lysis Kit, abm, Canada) for cDNA Extraction]; the DNA was stored at −20°C.

Quantitative reverse transcriptase PCR (qRT-PCR) was performed with a Roche LightCycler® 480 instrument and EvaGreen 2× qPCR MasterMix. The 20-μl reaction system contained 0.6 μl each of forward and reverse primers, 1 μl template cDNA, 10 μl EvaGreen 2× qPCR MasterMix, and 7.8 μl nuclease-free H_2_O. The primer sets used for these analyses are listed in Table 1. A no-template control was included during each qPCR experiment. The thermal cycling program started with 95°C for 10 min, followed by 40 cycles of 95°C for 15 s and 60°C for 1 min. After qPCR amplification, the comparative threshold method (ΔΔCt analysis) was applied to evaluate relative changes in gene expression. The experiment used GraphPad Prism 8 software for mapping (Conway et al., 2012). ΔΔCt = ΔΔCt, _sample_ – ΔΔCt, _reference_.

**Table 1:** Primers used in this study.

Gene	Sequence (5'–3')
*lasI*	Forward: CAGGTTTCCGGTTCGTGG Reverse: TTCCTTGCCGTGCAGAAG
*lasR*	Forward: TCTACCAGACGCGAAAGCAG Reverse: GTTTGCTGACCGGATGTTCG
*rhlI*	Forward: GTTCGACCATCCGCAAAC Reverse: ACGTCCTTGAGCAGGTAG
*rhlR*	Forward: GACCAGCAGAACATCTCC Reverse: CTGGGTCAGCAACTCGAT
*pqsH*	Forward: GAGACGCTGATCCTGTTC Reverse: CGATTCCCACTGACCAAG
*pqsR*	Forward: TTGATCGTCGCCAGGCTATC Reverse: TCGTTCTGCGATACGGTGAG
*lasB*	Forward: AACCGTGCGTTCTACCTGTT Reverse: CGGTCCAGTAGTAGCGGTTG
*phzH*	Forward: TGCGCGAGTTCAGCCACCTG Reverse: TCCGGGACATAGTCGGCGCA
*rpsL*	Forward: TGTGCTCTTGCAGGTTGTGA Reverse: TCGGCACTGCGTAAGGTATG

### Statistical Analysis

All assays were performed with three replicates and the values obtained are expressed as the mean ± SD. Differences in data were compared with the untreated control at each time-point and considered significant when *p* < 0.05 (^*^p < 0.05, ^**^p < 0.01, and ^***^p < 0.001) by one-way ANOVA with *t*-tests using GraphPad Prism 8 software.

## Results

### Synthesis of Str7410

Synthesis of compound Str7410 was accomplished in four steps from the commercially-available 1-bromoheptane (Scheme 1). Single substitution of *N*-tert-butoxycarbonyl-1,2-ethylenediamine with 1-bromoheptane generated intermediate 1. Removal of the Boc amino-protecting group followed by reaction with ethyl 2-bromoacetate afforded intermediate 3. Acidic hydrolysis twice afforded the desired compound Str7410.

### Effect of Compound Str7410 on *Vibrio harveyi* AI-2 QS

We used *V. harveyi* BB170 (a *luxN* mutant) as a reporter strain to evaluate the AI-2 QS inhibitory activity of test compounds. The IC_50_ values of compounds were tested in terms of *V. harveyi* BB170 bioluminescence. Bromo-furanone C56 (Zang et al., 2009) was used as the positive control. As shown in Table 2, Str7410 had the best inhibitory effect on AI-2 QS of *V. harveyi* BB170 (IC_50_ = 0.3724 ± 0.1091 μM). Based on this, compound Str7410 was selected for further research.

**Table 2:** Anti-*Vibrio harveyi* BB170 autoinducer-2 (AI-2) quorum sensing (QS) activities of test compounds and its structure.

Number	Structure	IC_50_ (μM)
C56	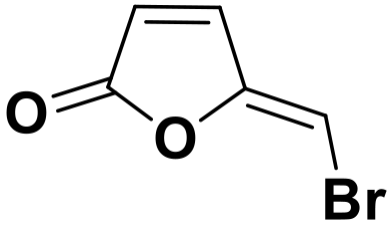	4.1 ± 1.2
Str6793	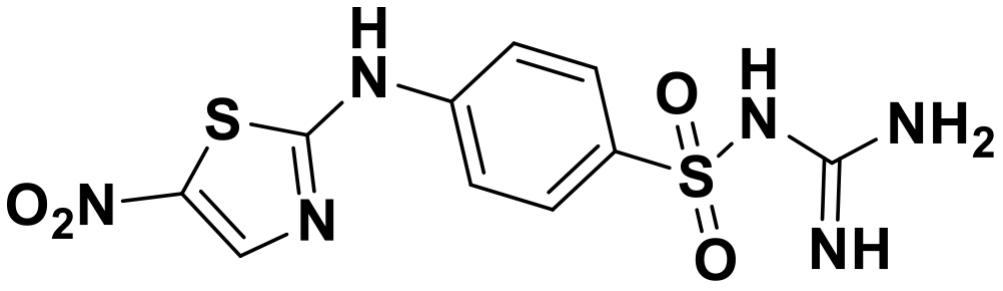	30.1 ± 4.5
Str7410	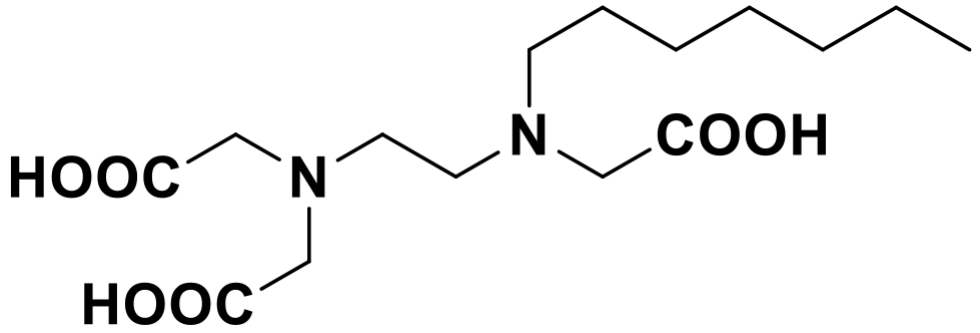	0.4 ± 0.1
Str7369	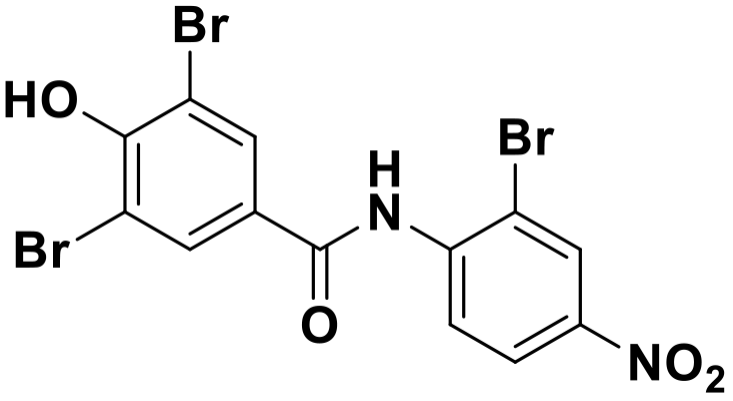	1.8 ± 1.1
Str629	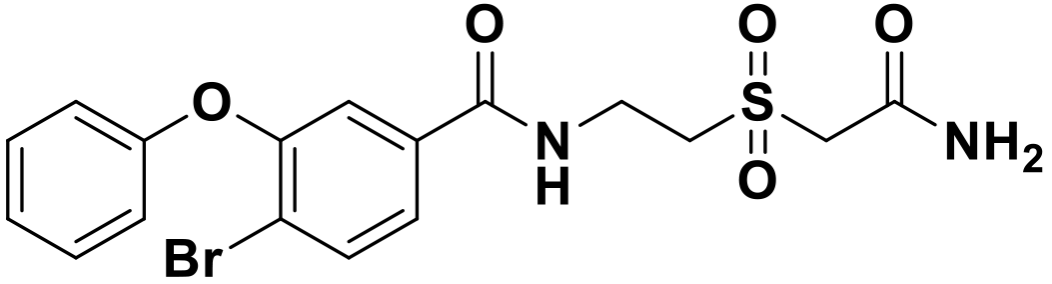	29.4 ± 8.8
Str811	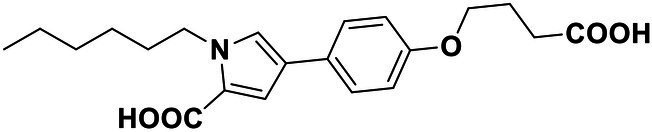	11.5 ± 5.5
Str1107	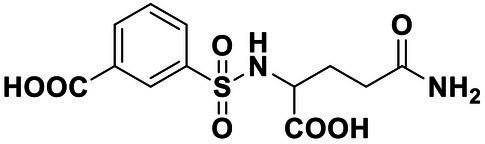	141.8 ± 23.5
Str120	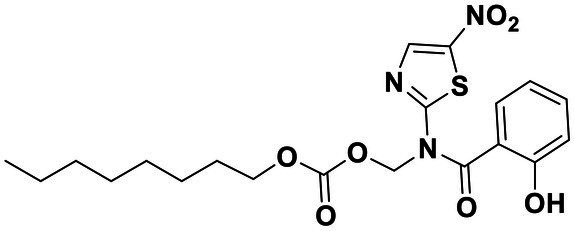	1.8 ± 1.1
StrOMe-165-10	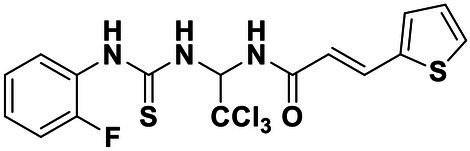	64.4 ± 9.6
Str3533	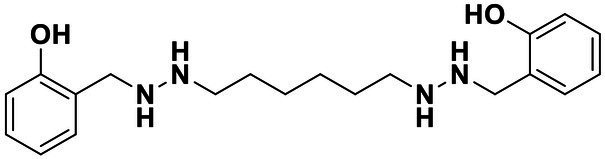	30.1 ± 4.5
Str5036	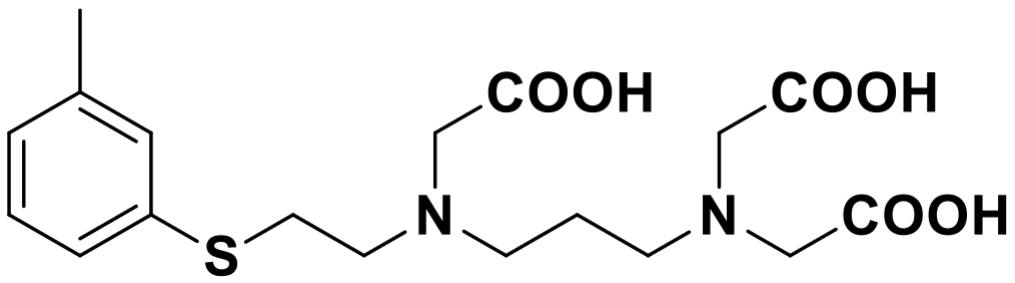	41.0 ± 22.9
Str5015	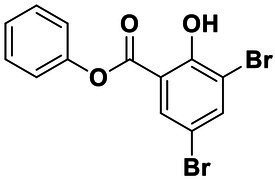	60.9 ± 8.5
StrOMe-780-1	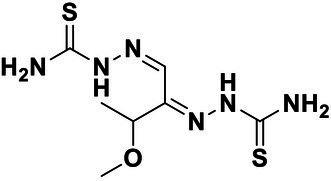	64.5 ± 10.2
Str5722	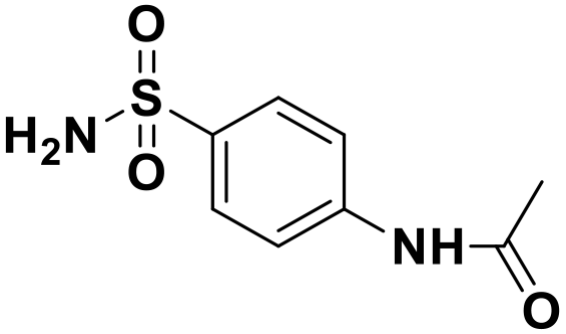	1162.0 ± 476.1
Str6467	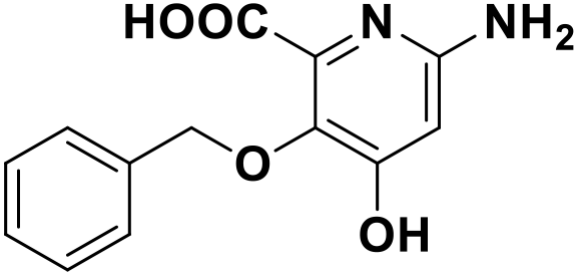	92.1 ± 30.9

Values are presented as the mean ± SD, *n* = 3.

### Effect of Compound Str7410 on Bacterial Growth

**Figure 1:**
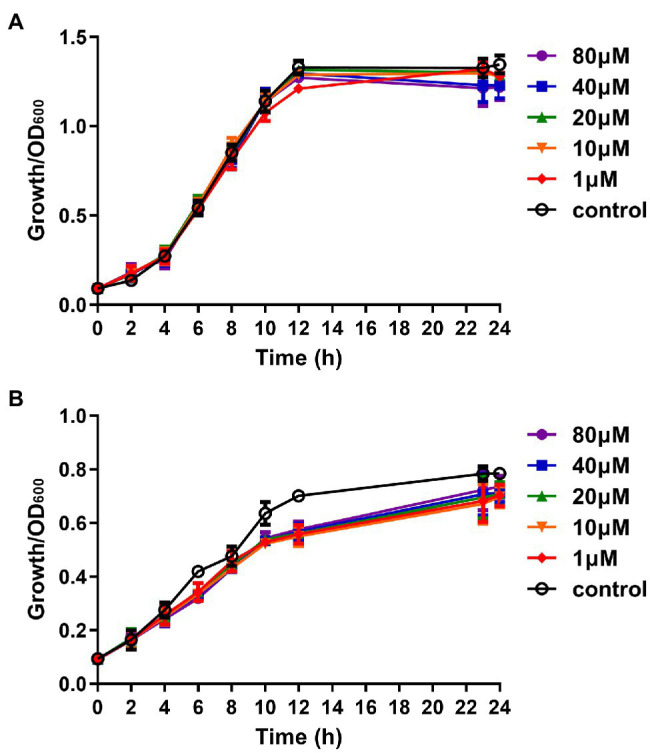
Effect of compound Str7410 (1–80 μM) on the growth of *Pseudomonas aeruginosa* PAO1 and *Staphylococcus aureus* ATCC 25923. **(A)** *Pseudomonas aeruginosa* PAO1 growth curves. **(B)** *Staphylococcus aureus* ATCC 25923 growth curves. Values are presented as the mean ± SD (*n* = 3).

The main difference between QSIs and antibiotics is that QSIs inhibit the production of virulence factors without killing pathogenic bacteria, so that the bacteria do not develop drug-resistance mutations (Allen et al., 2014). Therefore, we analyzed the effect of the putative QSI compound Str7410 on the growth of *P. aeruginosa* PAO1 and *S. aureus* ATCC 25923. As Figure 1 shows, compound Str7410 affected the growth of neither bacterium.

### Effect of Compound Str7410 on Biofilm Formation

**Figure 2:**
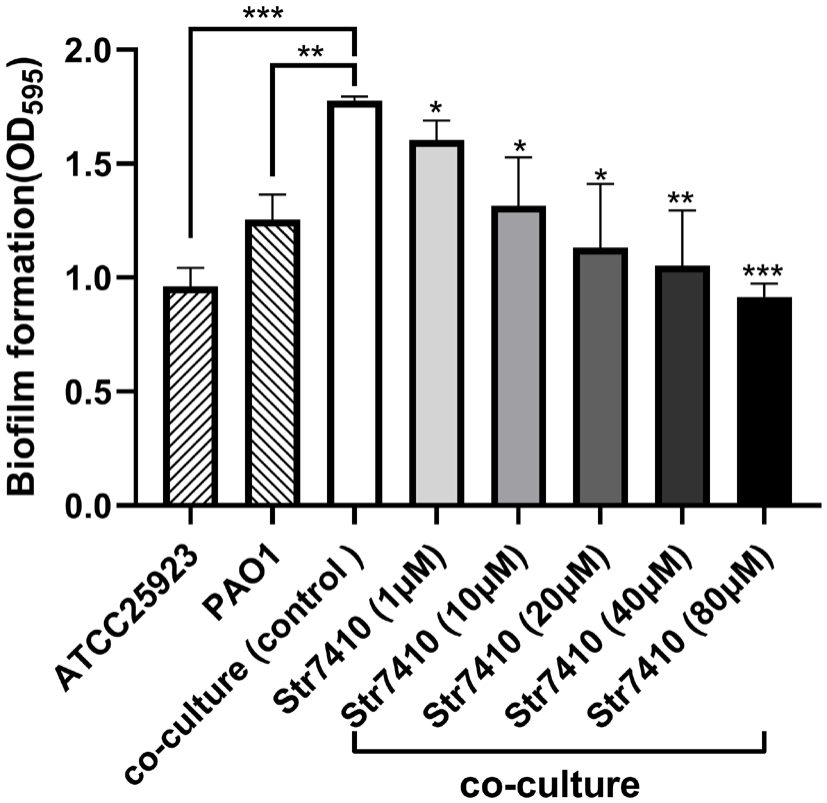
Crystal violet assay of biofilm formation by *Pseudomonas aeruginosa* PAO1 and *Staphylococcus aureus* ATCC 25923 alone and in co-culture after 24 h, and the inhibitory effect of compound Str7410 on biofilm formation by the mixed culture. Values are the mean ± SD (*n* = 3). Differences in data were compared with the untreated control at each time-point and considered significant when *p* < 0.05 (^*^p < 0.05, ^**^p < 0.01, and ^***^p < 0.001) by one-way ANOVA with *t*-tests.

Bacterial resistance to antibiotics is related to the formation of biofilms (Kalia, 2013). We used the crystal violet assay to evaluate the effect of compound Str7410 on formation of biofilms by *P. aeruginosa* PAO1 and *S. aureus* ATCC 25923 mixed in equal proportion. As shown in Figure 2, significantly more biofilm was formed by the mixture of species than by either species alone. When compound Str7410 was added to the co-culture, the formation of biofilm was significantly reduced [range 9.75% (*p* < 0.05)–48.40% (*p* < 0.001)]. The inhibitory effect of compound Str7410 was concentration-dependent. When the concentration of Str7410 was 40 μM, the inhibition rate of biofilm formation was 40.58% (*p* < 0.01).

### Effect of Compound Str7410 on the Antibiotic Susceptibility of Biofilm Cells

First, the MIC and MBIC values of antibiotics for single and mixed bacteria were determined when *P. aeruginosa* PAO1 was cultured alone or in co-culture with *S. aureus* ATCC 25923. As shown in Table 3, compared with planktonic cells, biofilm cells showed significantly increased resistance to antibiotics. The MBIC values of MEPM against biofilm cells increased 32 and 16-fold, respectively, compared with the MIC values for planktonic cells, when *P. aeruginosa* PAO1 was cultured alone and co-cultured with *S. aureus* ATCC 25923. Moreover, planktonic cells or biofilm cells from co-cultures of *P. aeruginosa* PAO1 and *S. aureus* ATCC 25923 showed significantly increased resistances to some antibiotics (ceftazidime, colistin sulfate, ciprofloxacin, and MEPM) compared with that of *P. aeruginosa* PAO1 monoculture. These data indicate that cultures of multiple bacterial species and the formation of biofilms can increase resistance to antibiotics. We also evaluated the MBEC of the compound Str7410 against the formed biofilm. As shown in Supplementary Table S1, the compound Str7410 had no eradication effect on the formed biofilm.

**Table 3:** Minimal inhibitory concentration (MIC) and minimum biofilm inhibitory concentration (MBIC) of antibiotics toward planktonic and biofilm cells of *Pseudomonas aeruginosa* PAO1 alone or in co-culture with *Staphylococcus aureus* ATCC 25923.

Antibiotic	PAO1		Mixed species	
	Planktonic cells MIC (μg/ml)	Biofilm cells MBIC (μg/ml)	Planktonic cells MIC (μg/ml)	Biofilm cells MBIC (μg/ml)
Tobramycin base	2	8	2	8
Ceftazidime	1	2	8	8
Amikacin	4	16	4	16
Colistin sulfate	4	64	>64	>64
Ciprofloxacin	0.08	0.25	0.25	0.5
Meropenem trihydrate	0.25	8	0.5	8

Data were obtained from three replicate experiments, *n* = 3.

**Figure 3:**
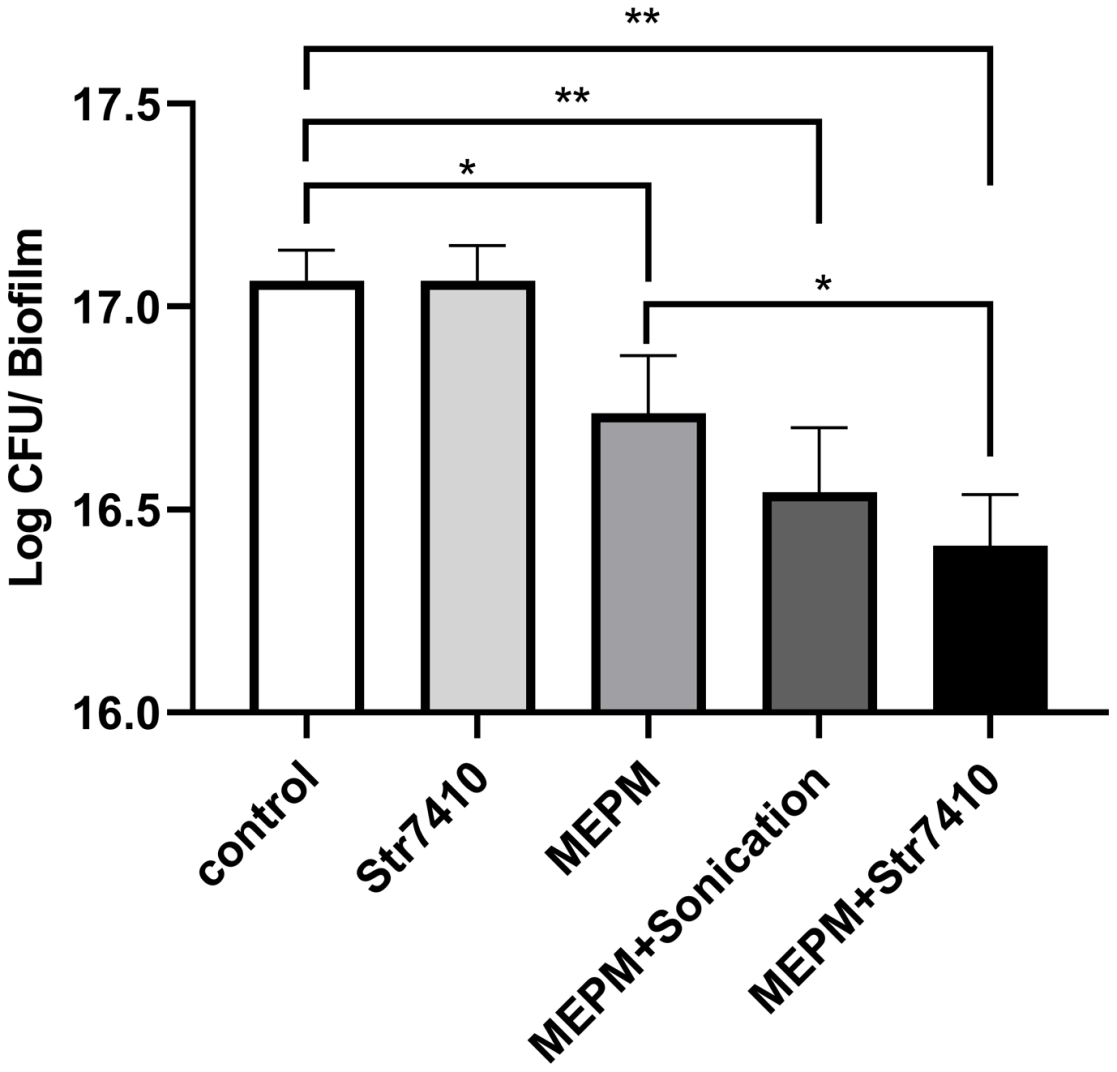
Effect of compound Str7410 on the number of cells [determined as log colony-forming units (CFU)] in biofilms formed from mixed *Pseudomonas aeruginosa* PAO1 and *Staphylococcus aureus* ATCC 25923 cells. Treatments: Str7410 (10 μM) alone, meropenem trihydrate (MEPM, 0.5 μg/ml) alone, and MEPM combined with sonication or Str7410. Values are presented as the mean ± SD (*n* = 3). Differences in data were compared with the untreated control at each time-point and considered significant when *p* < 0.05 (^*^p < 0.05, and ^**^p < 0.01) by one-way ANOVA with *t*-tests.

Next, we evaluated the effects of compound Str7410 on the susceptibility of mixed-species biofilms to antibiotics. We tested the inhibitory effects of MEPM and compound Str7410 alone, and MEPM combined with compound Str7410. MEPM in combination with sonication was used as a reference treatment. As Figure 3 shows, compound Str7410 when applied alone had no inhibitory effect on biofilms of mixed *P. aeruginosa* PAO1 and *S. aureus* ATCC 25923 cells. Treatment with MEPM alone and MPEM combined with sonication significantly decreased the number of biofilm cells (*p* < 0.05 and *p* < 0.01, respectively). The combination of MEPM with Str7410 also had a significant inhibitory effect on biofilm cells (*p* < 0.01). Notably, compound Str7410 increased the inhibitory effect of MEPM toward biofilm cells (*p* < 0.05), and the level of inhibition by Str7410 + MEPM was greater than that by MEPM + sonication.

### Effect of Compound Str7410 on Survival of Infected *Caenorhabditis elegans*

We used wild-type *C. elegans* N2 as a model to evaluate the effect of compound Str7410 on survival rates following co-infection with *P. aeruginosa* PAO1 and *S. aureus* ATCC 25923 *in vivo*.

**Figure 4:**
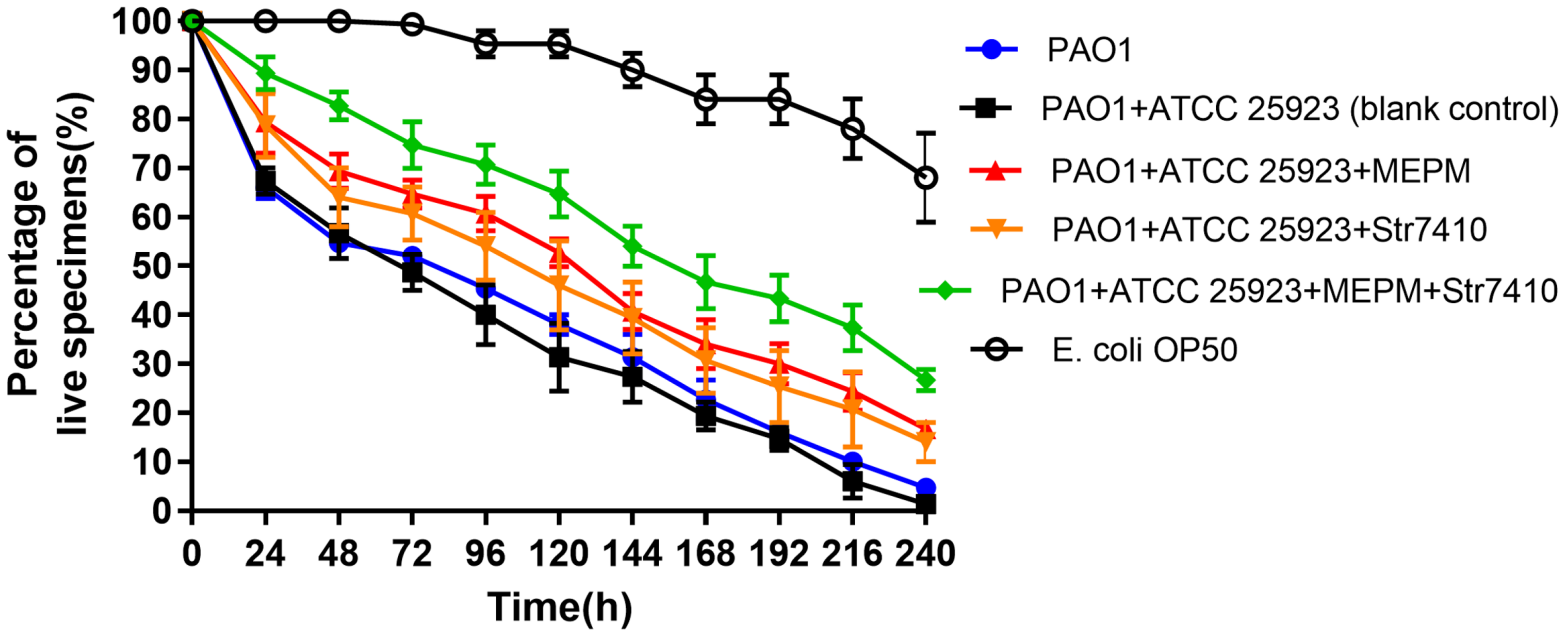
Effect of treatment with compound Str7410 (40 μM) and the antibiotic MEPM (0.5 μg/ml) alone or combination on survival of *Caenorhabditis elegans* N2 co-infected with *Pseudomonas aeruginosa* PAO1 and *Staphylococcus aureus* ATCC 25923. *Escherichia coli* OP50 was used as a negative control. Values are presented as the mean ± SD (*n* = 3).

After 240 h of incubation, the survival rate of nematodes decreased by 2.55% for *C. elegans* co-infected with *P. aeruginosa* PAO1 and *S. aureus* ATCC 25923 compared with that of nematodes infected with *P. aeruginosa* PAO1 alone (Figure 4). This indicates that the pathogenicity caused by multispecies infection was greater than that by a single bacterial species.

Compared with the positive control group co-infected by *P. aeruginosa* PAO1 and *S. aureus* ATCC 25923, the survival rate of the infected group treated with compound Str7410 (40 μM) increased by 12.67% (*p* < 0.05), and the survival rate of the infected group treated with MEPM (0.5 μg/ml) increased by 14.67% (*p* < 0.001). Furthermore, the survival rate of the group treated with the combination of Str7410 and MEPM increased by 12.67% (*p* < 0.05) and 10.67% (*p* < 0.05) compared with the Str7410-alone and MEPM-alone treatments, respectively (Figure 4). Although compound Str7410 inhibited the growth of neither *P. aeruginosa* PAO1 nor *S. aureus* ATCC 25923 (e.g., Figure 1), it decreased the pathogenicity of these bacteria by inhibiting interspecific QS. In addition, the combination of compound Str7410 with MEPM increased the therapeutic effect of MEPM.

### Effect of Compound Str7410 on Virulence Factors of *Pseudomonas aeruginosa* PAO1

**Figure 5:**
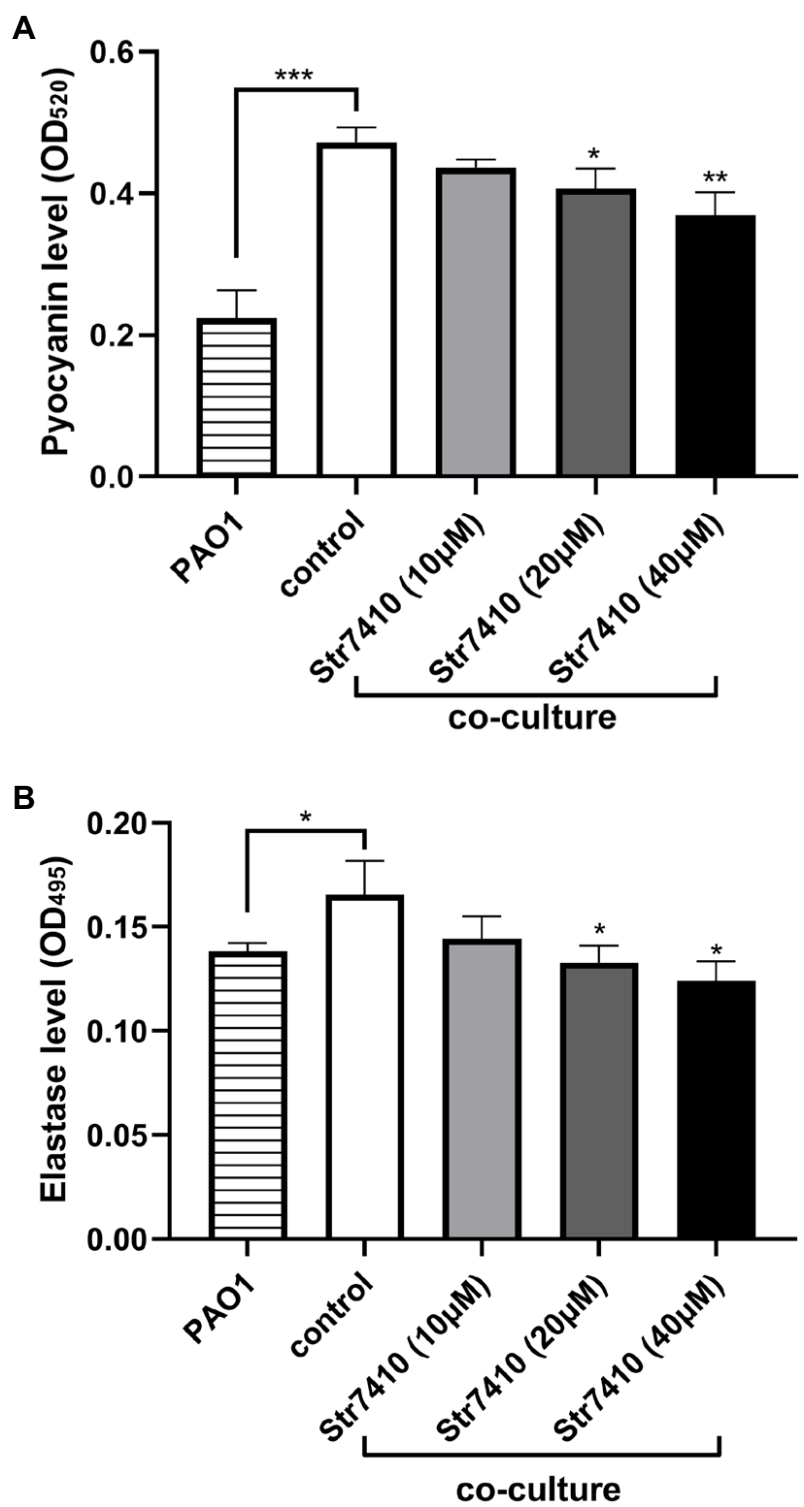
Production of virulence factors by *Pseudomonas aeruginosa* PAO1 in monoculture or co-culture with *Staphylococcus aureus* ATCC 25923, and the inhibitory effect of compound Str7410 on virulence factor production in co-culture. **(A)** Pyocyanin; **(B)** Elastase. Values are presented as mean ± SD (*n* = 3). Differences in data were compared with the untreated control at each time-point and considered significant when *p* < 0.05 (^*^p < 0.05, ^**^p < 0.01, and ^***^p < 0.001) by one-way ANOVA with *t*-tests.

The effect of Str7410 on virulence factors of *P. aeruginosa* PAO1 in multispecies infection was investigated. We quantitatively analyzed production of the virulence factors pyocyanin and elastase by *P. aeruginosa* PAO1 when this strain was cultured alone or co-cultured with *S. aureus* ATCC 25923. As Figure 5 shows, the production of pyocyanin and elastase was significantly increased (*p* < 0.001 and *p* < 0.05, respectively) when *P. aeruginosa* PAO1 was co-cultured with *S. aureus* ATCC 25923 compared with *P. aeruginosa* PAO1 monoculture. However, treatment with Str7410 significantly reduced the production of pyocyanin and elastase in co-cultures compared with the untreated controls; this effect of Str7410 was concentration dependent. When the concentration of Str7410 was 40 μM, the inhibition of production of pyocyanin and elastase was 22.07% (*p* < 0.01) and 24.85% (*p* < 0.05), respectively, compared with no treatment with Str7410.

### Effect of Compound Str7410 on Swarming Motility of *Pseudomonas aeruginosa* PAO1

**Figure 6:**
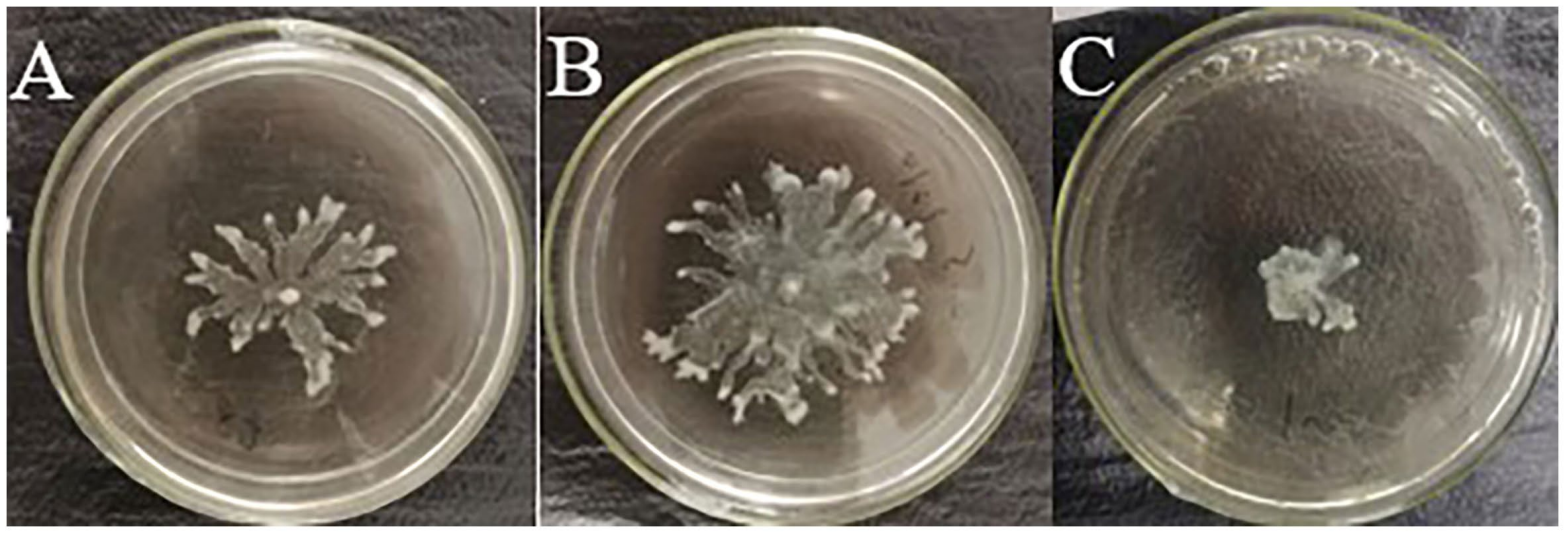
Effect of compound Str7410 on swarming motility of *Pseudomonas aeruginosa* PAO1. **(A)** Negative control (*P. aeruginosa* PAO1 alone). **(B)** Blank control (*P. aeruginosa* PAO1 with supernatant of *Staphylococcus aureus* ATCC 25923 culture). **(C)** Treatment group (*P. aeruginosa* PAO1 treated with supernatant of *S. aureus* ATCC 25923 culture and 10 μM Str7410).

Bacterial motility is important for pathogenicity and antibiotic resistance. Swarming motility, an important virulence factor of *P. aeruginosa*, is mainly regulated by QS-related genes, such as *lasB* and *pvdQ* (Tremblay and Deziel, 2008). Furthermore, *S. aureus* culture supernatant can promote the swarming motility of *P. aeruginosa* PAO1 under normoxia (Pallett et al., 2019). Similarly, in this study, culture supernatant of *S. aureus* ATCC 25923 promoted the swarming motility of *P. aeruginosa* PAO1 (Figures 6A,B). However, when compound Str7410 was added, the swarming motility was significantly inhibited (Figures 6B,C).

### Expression of QS-Related Genes in *Pseudomonas aeruginosa* PAO1 in Mixed Culture

**Figure 7:**
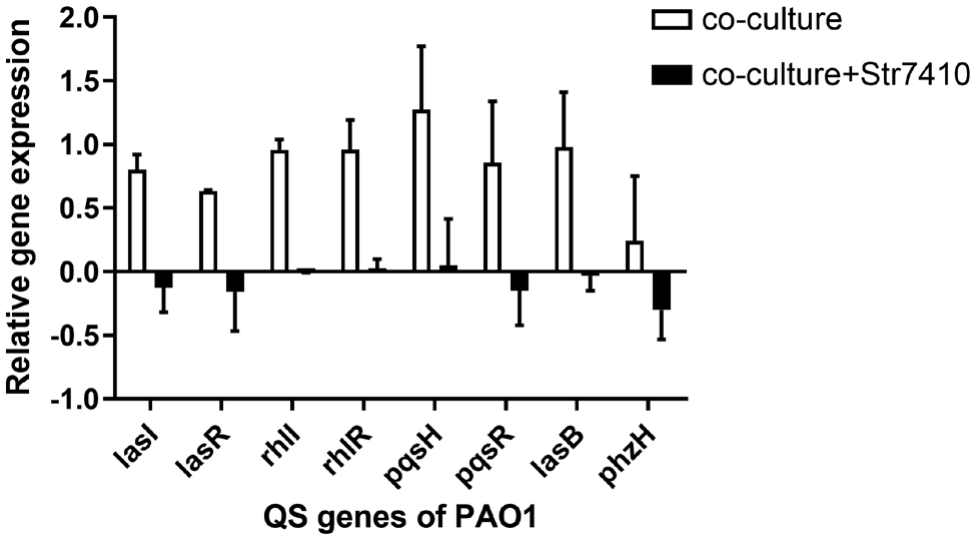
Expression on quorum sensing (QS) and virulence factor-related genes of *Pseudomonas aeruginosa* PAO1 when strain PAO1 was co-cultured with *Staphylococcus aureus* ATCC 25923 compared with PAO1 monoculture, and the effect of compound Str7410 (40 μM) on the expression of PAO1 QS and virulence factor-related genes in co-culture. Values are presented as the mean ± SD (*n* = 3).

From the data above, we found that *S. aureus* ATCC 25923 can increase the production of virulence factors and the swarming motility of *P. aeruginosa* PAO1 on co-culture of strains PAO1 and ATCC 25923. Compound Str7410 significantly inhibited this. These findings indicate that in multispecies infections, bacteria can enhance pathogenicity through their interactions, potentially *via* AI-2 QS systems. Therefore, we quantified the relative expression of QS-related genes of *P. aeruginosa* PAO1 in co-culture of *P. aeruginosa* PAO1 with *S. aureus* ATCC 25923. As shown in Figure 7, the expression of PAO1 QS-related genes (*lasI*, *lasR*, *rhlI*, *rhlR*, *pqsH*, and *pqsR*) and virulence factor-related genes (elastase, *lasB*; pyocyanin, and *phzH*) was significantly upregulated in the co-culture group compared with that in *P. aeruginosa* PAO1 monoculture. When Str7410 (40 μM) was added, the expression of all the strain PAO1 QS genes was significantly downregulated in co-culture compared with that in the absence of Str7410.

## Discussion

Bacterial infections, such as chronic wound infections and lung infections in patients with *CF*, are often caused by infection with multiple bacterial species (DeLeon et al., 2014; Nguyen and Oglesby-Sherrouse, 2016). *Pseudomonas aeruginosa* is a major opportunistic pathogen in hospitals, and nearly 80% of 18-year-old *CF* patients suffer chronic colonization and infection by *P. aeruginosa* (Duan et al., 2003). *Staphylococcus aureus* is another main bacterium in *CF* and chronic wounds. *Staphylococcus aureus* can interact with *P. aeruginosa* to promote the development of disease (Baldan et al., 2014). Studies demonstrated that *P. aeruginosa* and *S. aureus* compete with each other when co-cultured *in vitro*, and *P. aeruginosa* can produce 4-hydroxy-2-heptylquinoline-*N*-oxide (HQNO) through the *pqs* system to inhibit the aerobic respiration of *S. aureus* (Williams and Camara, 2009). In addition, virulence factors produced by *P. aeruginosa*, such as pyocyanin, have a significant inhibitory effect on the aerobic respiration and growth of *S. aureus* (Essar et al., 1990). However, although *P. aeruginosa* inhibits the growth of *S. aureus* in the planktonic state, a mixture of the two strains coordinates in the formation of biofilms and resistance to antibiotics. *Staphylococcus aureus* can avoid inhibition by *P. aeruginosa* by forming small colony variants, while the extracellular polymeric substances produced by *P. aeruginosa* can protect *S. aureus* from the killing effect of antibiotics (Atalla et al., 2011). Furthermore, *P. aeruginosa* and *S. aureus* co-culture promotes the production of virulence factors, which increase bacterial pathogenicity (Dalton et al., 2011; Orazi and O’Toole, 2017). Studies have indicated that QS mediated by AI-2 signaling molecules, a general communication system between bacteria, plays an important role in bacterial pathogenicity and drug resistance (Duan et al., 2003). In infectious disease, multiple species can coordinate with each other *via* QS, promoting bacterial pathogenicity and drug resistance. *Pseudomonas aeruginosa* can sense AI-2 signaling molecules produced by *Streptococcus mitis*, *Staphylococcus aureus*, and so on, leading to increased production of its own virulence factors and the formation of biofilms (Duan et al., 2003; Li et al., 2015; Wang et al., 2016b). Therefore, research into inhibitors of AI-2-mediated QS is a new strategy to target multispecies infections.

In previous study, we used the AI-2 signal molecule receptor LuxP as a target to screen 8,600 small molecule compounds from an in-house compound library using virtual screening techniques, and identified 14 AI-2 QSI compounds (Yuan, 2019). Wild-type *V. harveyi* senses AHL signal molecules as well as AI-2 signal molecules. To eliminate interference from AHLs, *V. harveyi* strain BB170 was used as the reporter here; this strain lacks the LuxN receptor for AHLs, but contains the LuxP receptor to sense AI-2 (Ren et al., 2001). As shown in Table 2, compound Str7410 had the best inhibitory effect on AI-2 QS by *V. harveyi* BB170 (IC_50_ = 0.3724 ± 0.1091 μM). Thus, we selected compound Str7410 as the test compound to evaluate its inhibitory effect on interspecific QS when *P. aeruginosa* PAO1 and *S. aureus* ATCC 25923 were co-cultured.

Because of abuse of antibiotics, bacterial drug resistance is increasing. In multibacterial infections, pathogenic bacteria can communicate with each other through AI-2 QS to promote pathogenicity and drug resistance (Roy et al., 2011). Biofilms, produced *via* QS, are an important reason for bacterial drug resistance. *Pseudomonas aeruginosa* can sense AI-2 produced by other bacteria, which regulates its production of virulence factors and formation of biofilms (Li et al., 2015; Wang et al., 2016b). Exogenous AI-2 can significantly increase *P. aeruginosa* biofilm formation (Li et al., 2017). However, an AI-2 QSI (D-ribose) affected the sensation of PAO1 to AI-2 and decreased the biomass of a mixed bacterial biofilm (Wang et al., 2016b). In this study, we found that *P. aeruginosa* PAO1 and *S. aureus* ATCC 25923 formed a mixed-species biofilm when co-cultured (Figure 2). Compound Str7410 inhibited QS between *P. aeruginosa* and *S. aureus*, thereby reducing the biofilm formation.

Nearly 80% of human infections are related to biofilms (Romero et al., 2008; Yan and Bassler, 2019). Meanwhile, the results of the present study showed that, compared with monoculture, co-culturing multiple species significantly reduced the effect of antibiotics (Table 3). In addition, compared with planktonic cells, biofilm formation significantly increased bacterial resistance to antibiotics (Table 3). Thus, the prevention and treatment of biofilms is a key concern of clinical staff and researchers (Stewart and Costerton, 2001). Studies have found that QSI can increase the susceptibility of pathogenic biofilm cells to antibiotics (Brackman et al., 2011; Furiga et al., 2015; Topa et al., 2020). Our study confirmed that compound Str7410 improved the susceptibility of a mixed-strain biofilm to the antibiotic MEPM, and that compound Str7410 can promote the inhibitory activities of antibiotics toward biofilm cells while having no effect on bacterial growth (Figure 1) and no eradication effect on the formed biofilm (Supplementary Table S1). Therefore, combining QSI with antibiotics is a new, and potentially highly effective, strategy for effective use of antibiotics against biofilm cells.

The nematode *C. elegans* is often used in *in vivo* models to study the pathogenicity of bacteria, for example, *Serratia marcescens*, *P. aeruginosa*, and *Staphylococcus aureus* (Powell and Ausubel, 2008; Brooks et al., 2009). The pathogenicity of co-infection with *P. aeruginosa* PAO1 and *S. aureus* ATCC 25923 toward *C. elegans* N2 was greater than that of either bacterial species alone. Compound Str7410 decreased the pathogenicity of the bacteria by inhibiting interspecific QS, thereby increasing the survival rate of infected nematodes (Figure 4). Thus, compound Str7410 can achieve a therapeutic effect in multispecies infections. Moreover, the combination of compound Str7410 with MEPM significantly increased the therapeutic effect of MEPM in infected nematodes.

We also evaluated the effect of compound Str7410 on the inhibition of interspecies QS *via* analysis of virulence factors of *P. aeruginosa*. Pyocyanin is a blue–green phenazine pigment, which has a number of physiological roles, such as facilitating biofilm development and influencing colony formation, and it is also one of the causes of pulmonary infections caused by *P. aeruginosa* (Wilson et al., 1988; Dietrich et al., 2006; Zhu et al., 2019). *Pseudomonas aeruginosa* regulates the production of pyocyanin through two QS pathways, the *rhl* and *pqs* systems (Diggle et al., 2003). Elastase destroys cellular tissues in infected hosts (Ben Haj Khalifa et al., 2011). Elastase is regulated by the QS-related *lasB* gene. Bacterial motility plays a critical role in pathogenicity and antibiotic resistance. *Pseudomonas aeruginosa* swarming motility is mainly regulated by QS-related genes such as *lasB* and *pvdQ* (Tremblay and Deziel, 2008). *Pseudomonas aeruginosa* can sense exogenous AI-2, which enhances the production of the virulence factors pyocyanin and elastase (Li et al., 2015). The peptidoglycan produced by *S. aureus* can also enhance the production of pyocyanin and elastase by *P. aeruginosa* (Korgaonkar et al., 2013). Under normoxia, the supernatant of *S. aureus* can promote the swarming motility of *P. aeruginosa* PAO1 (Pallett et al., 2019). In this study, we confirmed that the amount of pyocyanin and elastase produced by *P. aeruginosa* PAO1 significantly increased when *P. aeruginosa* and *S. aureus* were co-cultured (Figure 5), and *S. aureus* ATCC 25923 culture supernatant promoted the swarming motility of strain PAO1 (Figure 6B). Compound Str7410 significantly inhibited the virulence factor production and swarming motility of *P. aeruginosa* PAO1 during co-culture of *P. aeruginosa* PAO1 and *S. aureus* ATCC 25923 (Figures 5, 6C). We suggest that compound Str7410 reduced the production of virulence factors and swarming motility by inhibiting the sensation of *P. aeruginosa* PAO1 to AI-2 signaling molecules.

As a general QS system possessed by both Gram-positive and Gram-negative bacteria, AI-2 QS boosts the coordination and communication between bacteria in bacterial infection (Duan et al., 2003). Woods et al. (2018) found that there is an interaction between *P. aeruginosa* and *S. aureus* in co-cultured bacterial biofilms, and *S. aureus* can increase the expression of QS-related genes of *P. aeruginosa*. Moreover, the presence of exogenous AI-2 signal molecules can upregulate the expression of *P. aeruginosa* QS-related genes (Li et al., 2015). In this study (Figure 7), *S. aureus* ATCC 25923 significantly upregulated the expression of *P. aeruginosa* PAO1 QS-related genes (*lasI*, *lasR*, *rhlI*, *rhlR*, *pqsH*, and *pqsR*) and some QS target genes such as the virulence factor-related genes *lasB* and *phzH*. However, when compound Str7410 was added to the co-cultures, it reduced the pathogenicity of *P. aeruginosa* PAO1 by inhibiting the sensation of strain PAO1 to interspecific QS, thereby downregulating the expression of QS-related genes and the production of virulence factors.

In conclusion, this study focused on the inhibitory effects of a compound identified by virtual screening on the QS system of co-cultured Gram-positive bacterium *S. aureus* and Gram-negative bacterium *P. aeruginosa in vitro* and *in vivo*. Compound Str7410 was a potent AI-2 QSI. The combination of compound Str7410 with the antibiotic MEPM significantly increased the susceptibility of mixed-species-biofilm cells to the antibiotic. Preliminary mechanistic studies showed that Str7410 inhibited interspecific QS by inhibiting the AI-2 sensation of *P. aeruginosa* PAO1 and downregulating the expression of QS-related genes. This study provides a new strategy for the discovery of antibiotics in theory and a strategy for the clinical treatment of multidrug-resistant bacterial infections in practice.

## Data Availability Statement

The original contributions presented in the study are included in the article/Supplementary Material; further inquiries can be directed to the corresponding authors.

## Author Contributions

FL and JX conceived and designed the experiments. KJ, YX, BY, YY, MZ, RL, HW, LW, and YZ performed the experiments. KJ and YX created the figures, analyzed the data, and wrote the manuscript. All authors contributed to the article and approved the submitted version.

## Funding

This study was supported by the National Science and Technology Major Project of China (2018ZX09711003-003).

## Conflict of Interest

The authors declare that the research was conducted in the absence of any commercial or financial relationships that could be construed as a potential conflict of interest.

## Publisher’s Note

All claims expressed in this article are solely those of the authors and do not necessarily represent those of their affiliated organizations, or those of the publisher, the editors and the reviewers. Any product that may be evaluated in this article, or claim that may be made by its manufacturer, is not guaranteed or endorsed by the publisher.
