# Supplementary material for: Effect of Autoinducer-2 Quorum Sensing Inhibitor on Interspecies Quorum Sensing
Source: Front Microbiol. 2022 Mar 28;13:791802. doi: 10.3389/fmicb.2022.791802 (PMC8996156; doi:10.3389/fmicb.2022.791802)

**^1^H ,^13^C spectra and HRMS of compounds synthesized in this work**

**Tert-butyl (2-(heptylamino)ethyl)carbamate (1)**


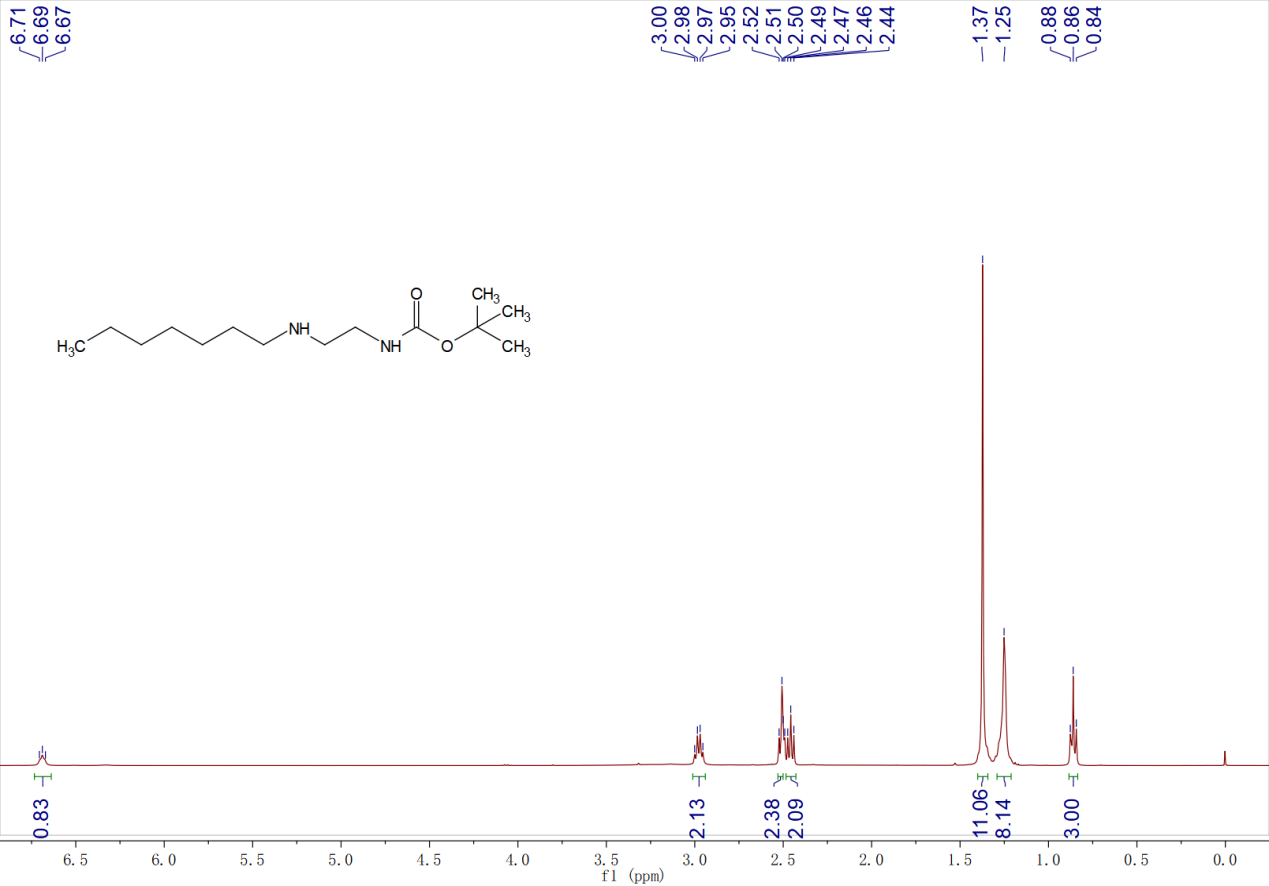


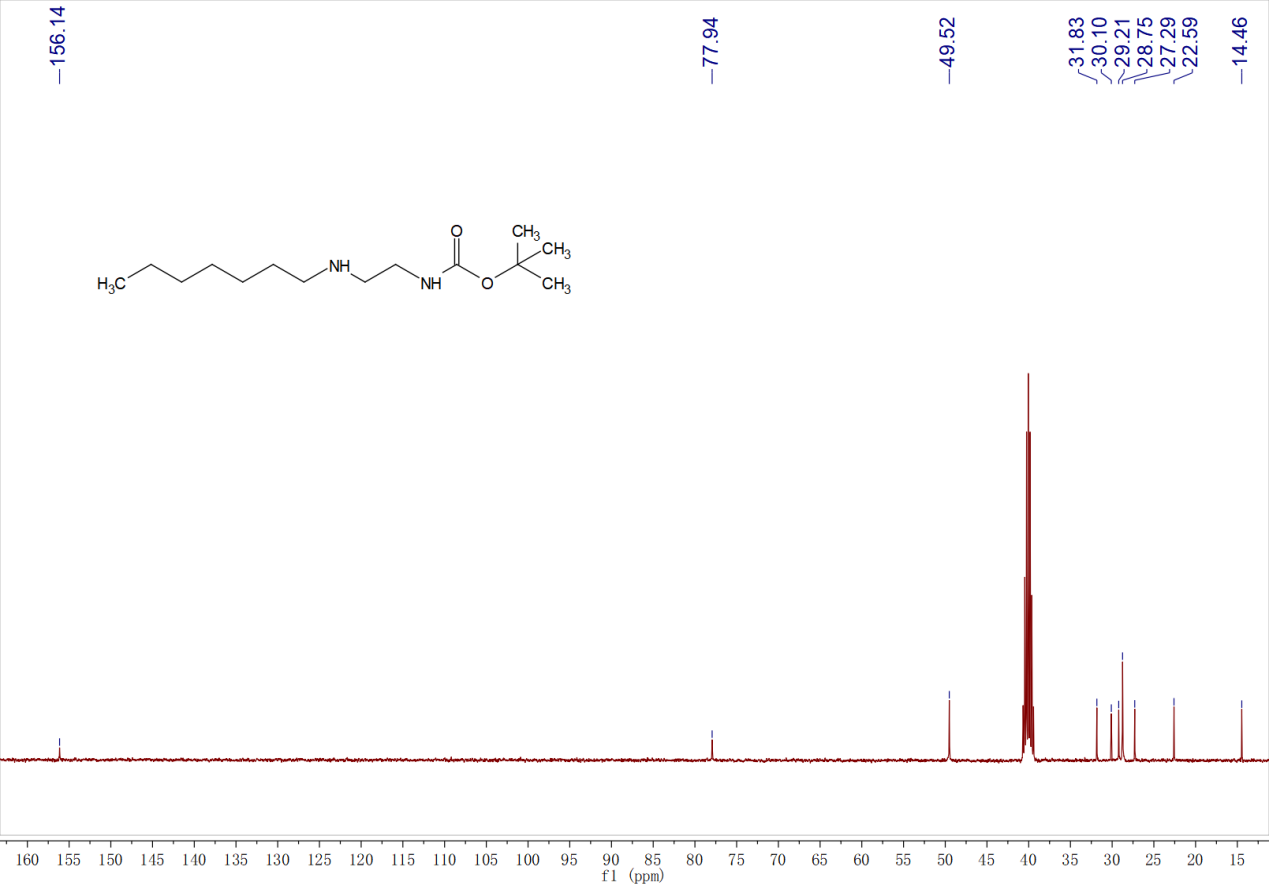


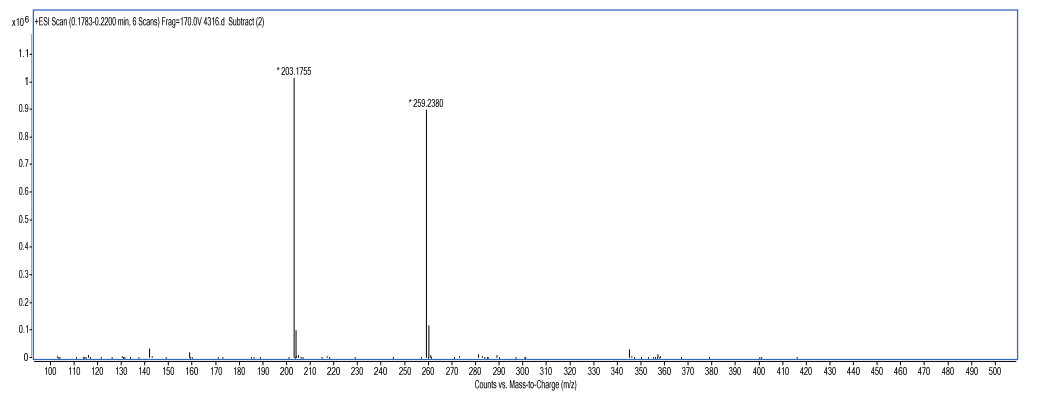


**Diethyl 2,2'-((2-((2-ethoxy-2-oxoethyl)(heptyl)amino)ethyl)azanediyl)diacetate (3)**

**
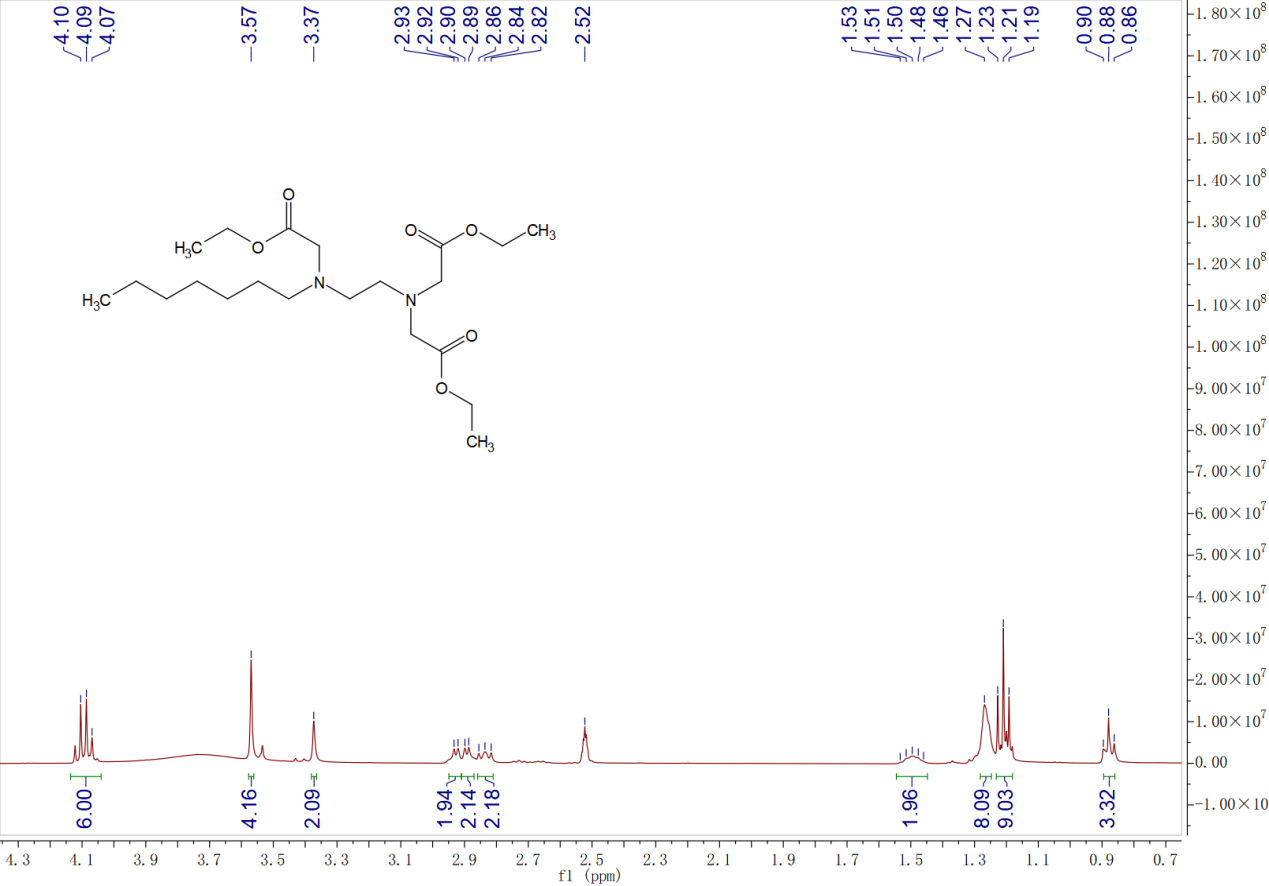
**

**
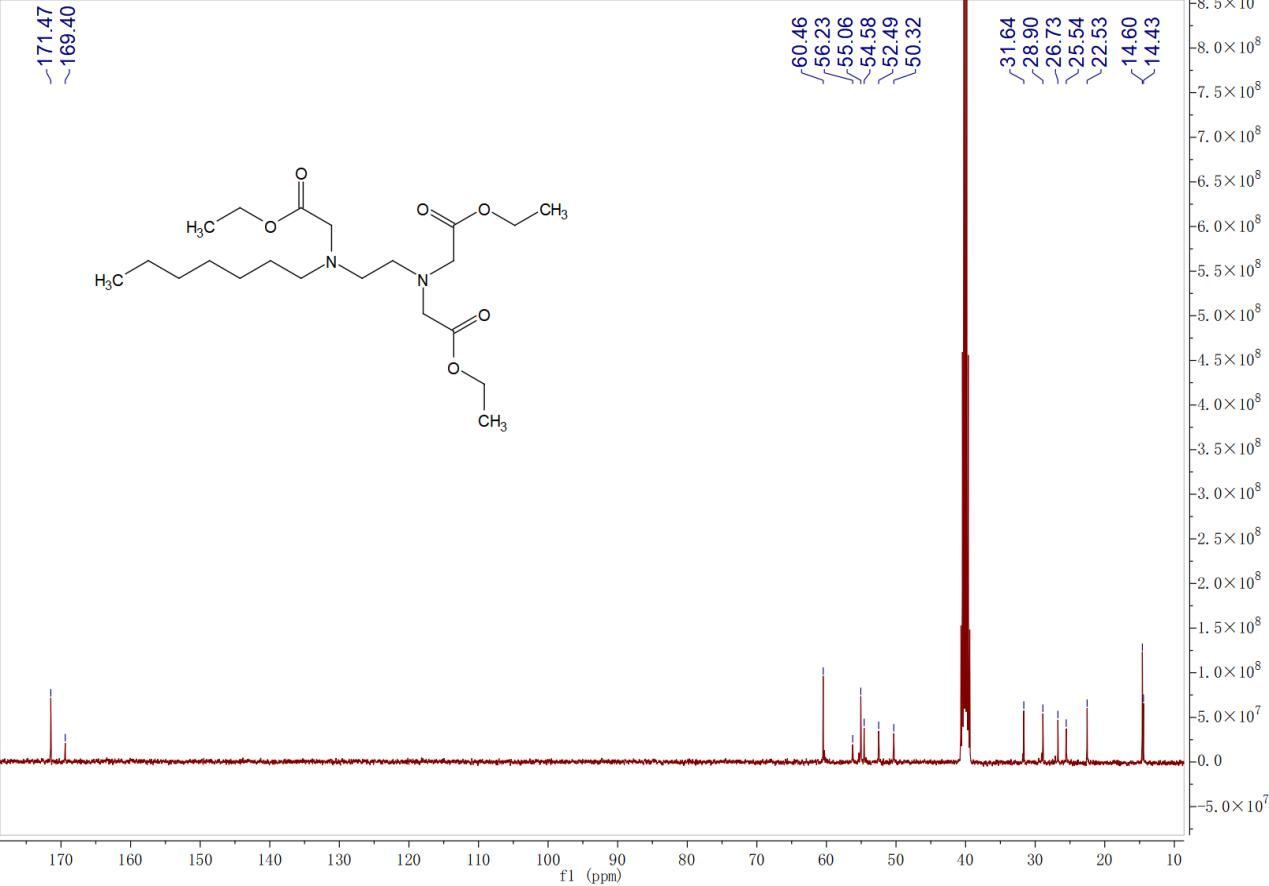
**


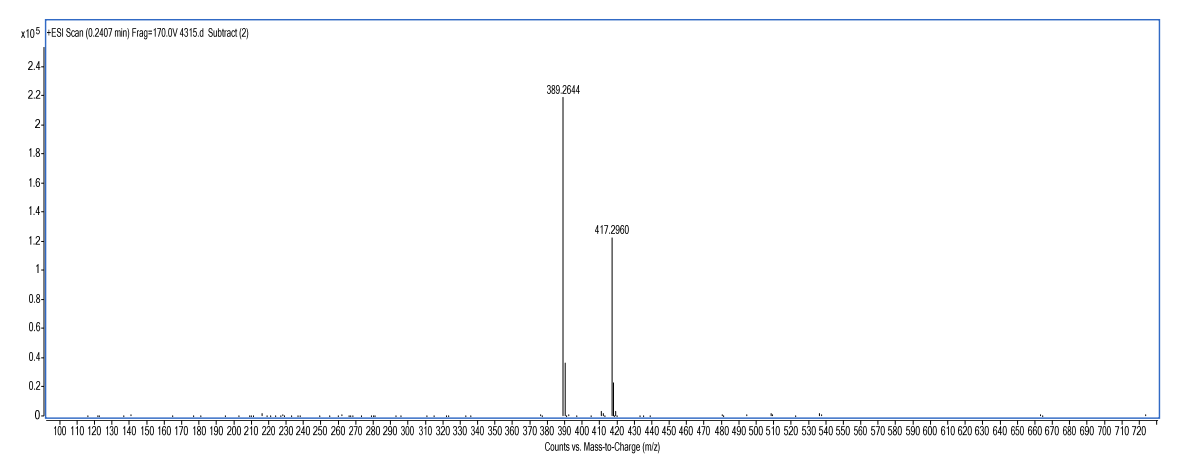


**2,2'-((2-((Carboxymethyl)(heptyl)amino)ethyl)azadiyl)diacetic acid (4)**


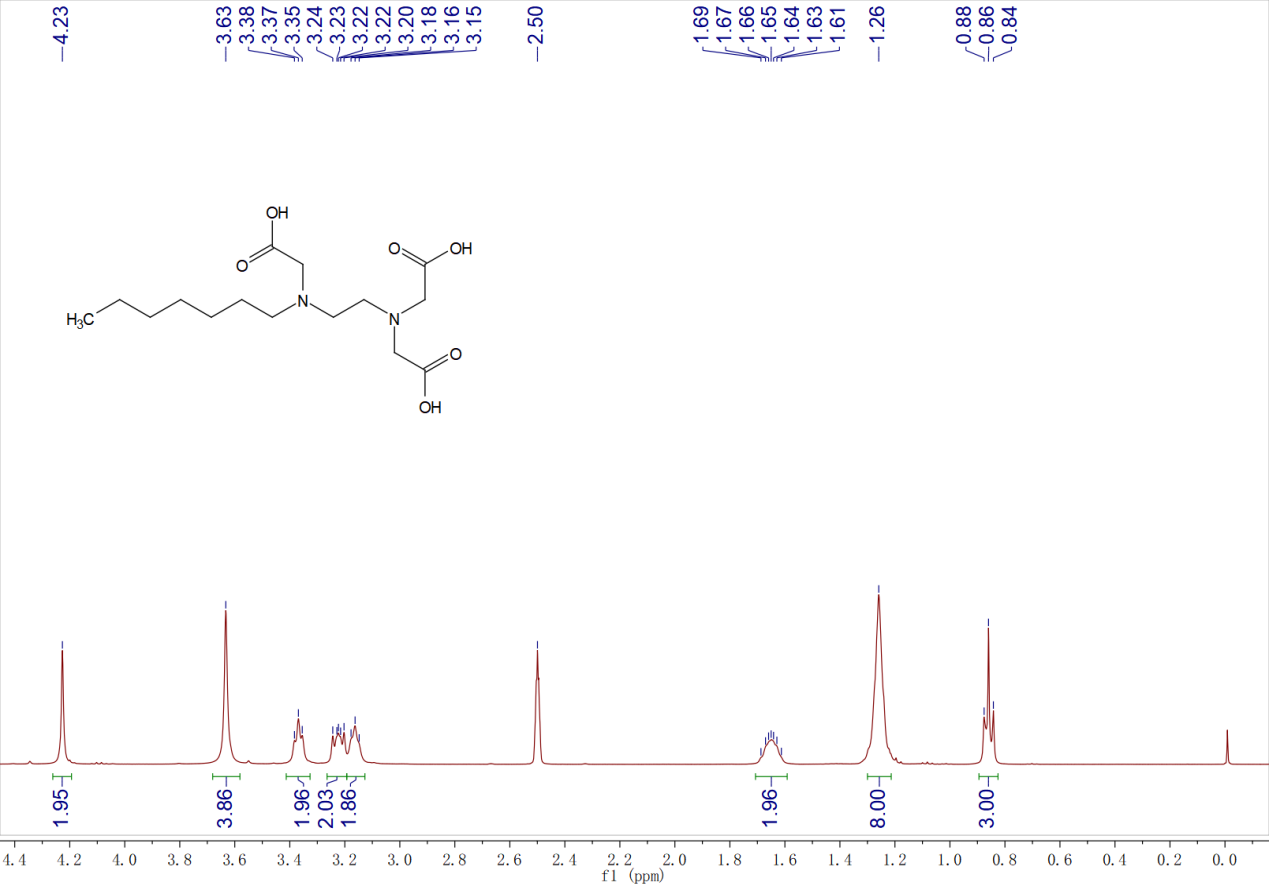


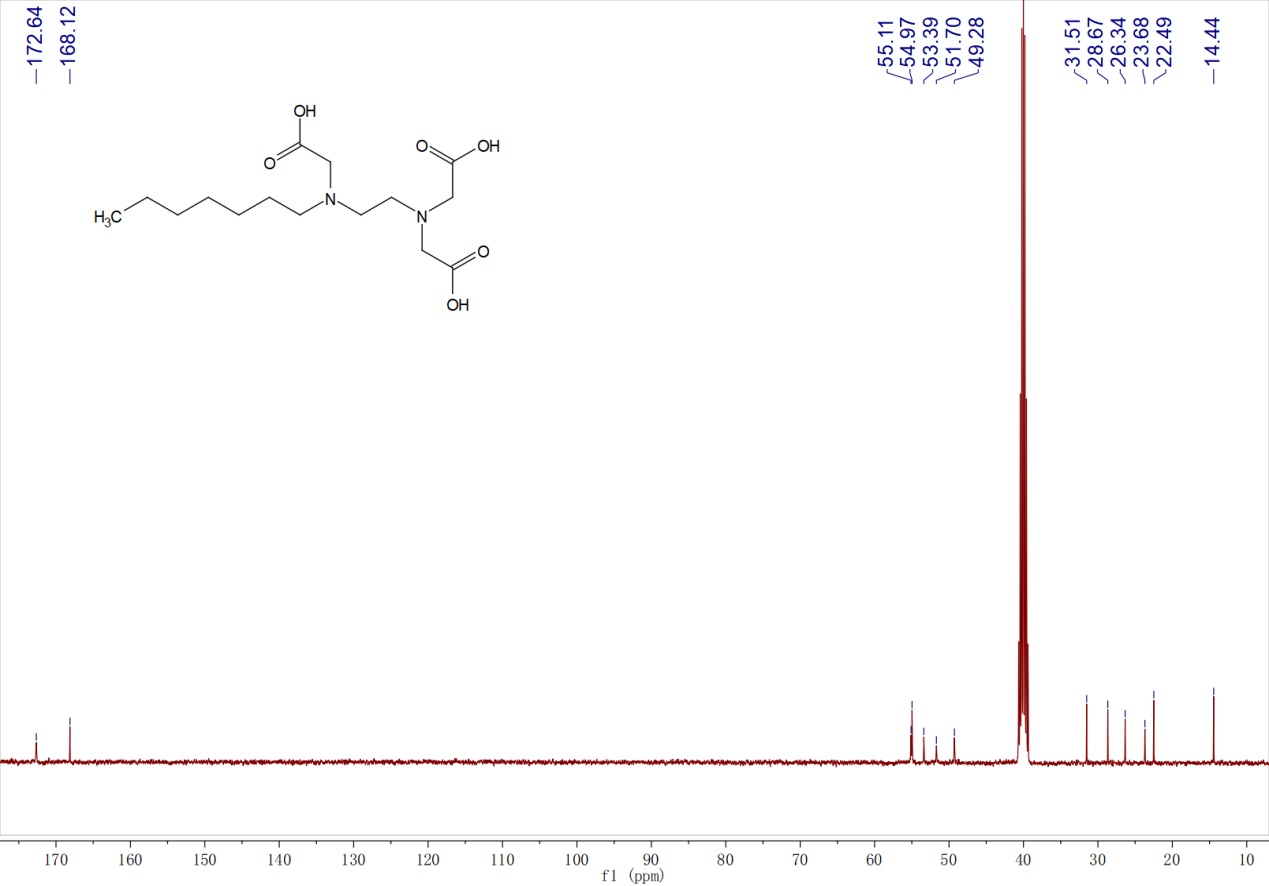


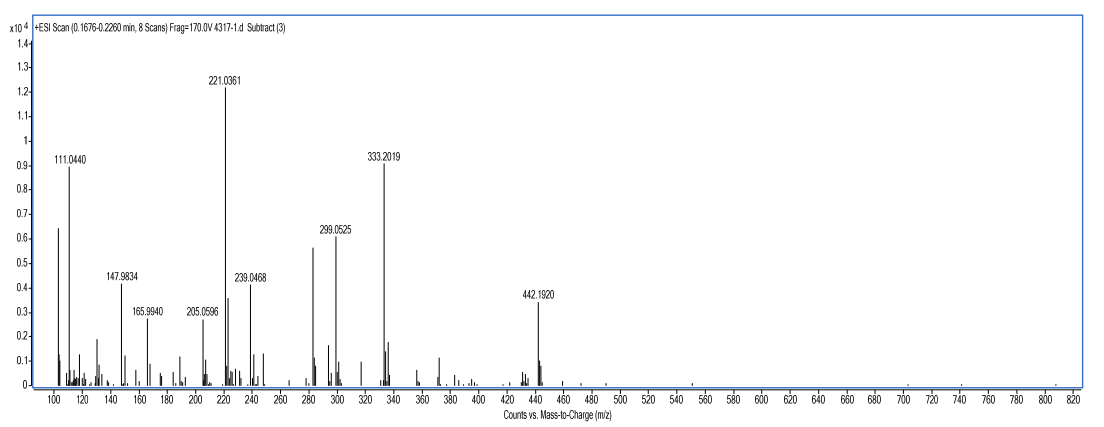

Supplement: Supplementary file 1 [file Data_Sheet_1.docx]
