# Supplementary material for: Effect of Autoinducer-2 Quorum Sensing Inhibitor on Interspecies Quorum Sensing
Source: Front Microbiol. 2022 Mar 28;13:791802. doi: 10.3389/fmicb.2022.791802 (PMC8996156; doi:10.3389/fmicb.2022.791802)
Supplement: Supplementary file 2 [file Table_1.docx]

TABLE S1. MBEC of compound Str7410 toward biofilms of *P. aeruginosa* PAO1 alone or in co-culture with *S. aureus* ATCC 25923

| Compound | PAO1 | Mixed species |
| --- | --- | --- |
|  | MBEC (μg/ml) | MBEC (μg/ml) |
| Str7410 | >1024 | >1024 |
